# Repurposing Farnesol for Combating Drug-Resistant and Persistent Single and Polymicrobial Biofilms

**DOI:** 10.3390/antibiotics13040350

**Published:** 2024-04-11

**Authors:** Li Tan, Rong Ma, Tony Reeves, Adam J. Katz, Nicole Levi

**Affiliations:** 1 Department of Plastic and Reconstructive Surgery, Wake Forest University School of Medicine, Winston-Salem, NC 27157, USA; ltan@wakehealth.edu (L.T.); akatz@wakehealth.edu (A.J.K.); 2 Department of Internal Medicine, Section on Molecular Medicine, Wake Forest University School of Medicine, Winston-Salem, NC 27157, USA

**Keywords:** farnesol, *Staphylococcus aureus*, *Pseudomonas aeruginosa*, drug-resistant, persistent, biofilm

## Abstract

Biofilm-associated infections caused by drug-resistant and persistent bacteria remain a significant clinical challenge. Here we report that farnesol, commercially available as a cosmetic and flavoring agent, shows significant anti-biofilm properties when dissolved in ethanol using a proprietary formulation emulsion technique. Farnesol in the new formulation inhibits biofilm formation and disrupts established biofilms for Gram-positive *Staphylococcus aureus* and Gram-negative *Pseudomonas aeruginosa*, including their polymicrobial biofilms, and, moreover, kills *S. aureus* persister cells that have developed tolerance to antibiotics. No resistance to farnesol was observed for *S. aureus* after twenty continuous passages. Farnesol combats biofilms by direct killing, while also facilitating biofilm detachment. Furthermore, farnesol was safe and effective for preventing and treating biofilm-associated infections of both types of bacteria in an ex vivo burned human skin model. These data suggest that farnesol in the new formulation is an effective broad-spectrum anti-biofilm agent with promising clinical potential. Due to its established safety, low-cost, versatility, and excellent efficacy—including ability to reduce persistent and resistant microbial populations—farnesol in the proprietary formulation represents a compelling transformative, translational, and commercial platform for addressing many unsolved clinical challenges.

## 1. Introduction

Biofilms, the structured community of bacteria embedded in a self-produced polymeric matrix and adherent to surfaces or tissues [1], are the root cause of over 80% of all microbial infections in humans [2]. Biofilm-related infections caused by drug-resistant pathogens, such as the Gram-positive bacterium *Staphylococcus aureus* and the Gram-negative bacterium *Pseudomonas aeruginosa*, are extremely difficult to treat with antibiotics. Biofilm-encased cells can be up to 1000-fold more resistant to various antibiotics than their planktonic counterparts [2]. Currently, antibiotic resistance is spreading much faster than the introduction of new drugs into clinical practice [3], triggering a significant public health crisis. Moreover, persister cells, a minor variant bacterial subpopulation existing in a dormant and drug-tolerant state, are further attributed to the recalcitrance and recurrence of biofilm-associated infections [4]. In addition, polymicrobial biofilms composed of both *S. aureus* and *P. aeruginosa* have been frequently identified in chronic wounds, including burns, non-healing surgical site infections and ulcers [5,6,7], resulting in delayed wound healing compared to single-species biofilms, further frustrating clinical strategies for biofilm treatment [7,8].

Farnesol is an acyclic sesquiterpene alcohol with a delicate sweet odor found in plants and mammals, including humans. It was approved by the Food and Drug Administration with a generally recognized as safe (GRAS) designation, and has wide applications in the food, cosmetic and perfume industries [9,10,11]. Farnesol was first discovered in the fungus *Candida albicans* as a quorum sensing molecule to inhibit filamentation and biofilm formation [12]. Since then, farnesol has been found to be very versatile, with antimicrobial, antitumor, cardioprotective, hepatoprotective, and neuroprotective activities [13,14,15,16,17]. Farnesol was found to be effective at hindering the growth of Gram-positive bacteria, but was less effective against the growth of Gram-negative bacteria; moreover, the published literature to date suggests that farnesol has only minor effects on disrupting established biofilms for both Gram-positive and negative bacteria [9,18,19,20,21]. Thus, farnesol alone was not proposed as an effective antimicrobial agent [22]. Nevertheless, farnesol has been considered to be a promising adjuvant for conventional antibiotics due to its synergistic effect [22,23,24,25].

Since farnesol is highly water insoluble [26], which reduces its bioavailability as an antibacterial agent by itself, it has previously been dissolved in various solvents, such as dimethyl sulfoxide, methanol, or Tween 80 (Table S1). Due to the toxicity of these vehicles themselves, the highest achievable doses of farnesol without confounding vehicle killing have been limited. We have recently developed a proprietary formulation emulsion technique using ethanol as a vehicle and then interfacing the farnesol in ethanol with aqueous media. Farnesol dissolving in ethanol could reach the highest stock concentration of 30 mg/mL, which allowed us to test the effect of solubilized farnesol on bacterial biofilms using a dose range much wider than those in previous studies (Table S1). The novel formulation of farnesol proved to be safe and highly effective against biofilms of drug-resistant bacteria such as *S. aureus* and *P. aeruginosa*, both in vitro and ex vivo.

## 2. Results

### 2.1. Farnesol Is Highly Effective against S. aureus Biofilms

**Figure 1:**
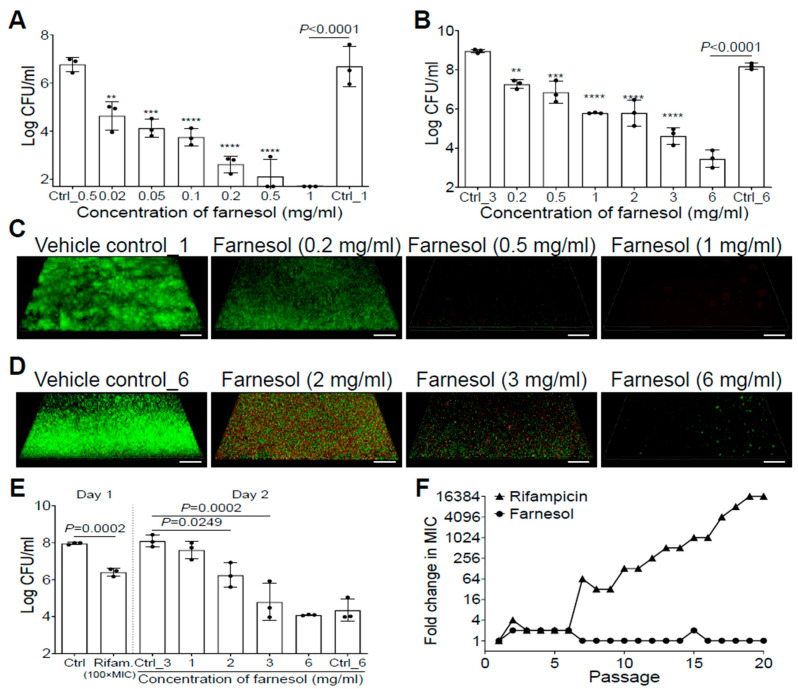
Farnesol inhibits biofilm formation, disrupts established biofilms, and kills persister cells of *S. aureus* Xen29 without inducing resistance. (**A**) Elimination of biofilm formation of *S. aureus* Xen29 by farnesol 24 h after incubation in NB1 in plasma-precoated wells. (**B**) Disruption of established 24 h old biofilms of *S. aureus* Xen29 followed by 24 h exposure of farnesol. (**C**,**D**) Three-dimensional merged images of Live/Dead viability of *S. aureus* Xen29 biofilms after 24 h incubation (developing biofilm) (**C**) or 24 h exposure of 24 h old established biofilms (**D**) to NB1 containing farnesol. Biofilms were stained with both SYTO^®^ 9 (green fluorescence for live cells) and propidium iodide (red fluorescence for dead cells). Scale bars, 20 μm. (**E**) Farnesol kills *S. aureus* Xen29 persister cells tolerant to rifampicin treatment. Biofilms of *S. aureus* Xen29 were treated for 24 h with 100× minimal inhibitory concentration (MIC) of rifampicin (Rifam.). Rifampicin was removed and persister cells were further exposed to NB1 containing 0 to 6 mg/mL of farnesol for an additional 24 h. (**F**) Resistance development of *S. aureus* Xen29 to farnesol, or the antibiotic rifampicin, respectively, during serial passaging in the presence of sub-MIC levels of antimicrobials. Data are fold changes (in log_2_) in MIC relative to the MIC of the first passage (16 µg/mL for farnesol; 8 ng/mL for rifampicin). The *X*-axis line in (**A**) represents the lower limit of detection (Log 50 ≈ 1.7). Data in (**A**,**B**,**E**) are expressed as the number of viable bacteria in log10 CFU per mL and was shown as mean ± SD (n = 3). Ctrl, control. ** *p* < 0.01, *** *p* < 0.001, and **** *p* < 0.0001. Ctrl_# represents the vehicle (ethanol) control corresponding to the same amount of farnesol in ethanol. Ctrl_0.5 = 1.7% of ethanol; Ctrl_1 = 3.3% of ethanol; Ctrl_3 = 10% of ethanol; and Ctrl_6 = 20% of ethanol.

Farnesol inhibited *S. aureus* Xen29 biofilm formation in a dose-dependent manner, and completely eradicated the bacteria at a concentration of 1 mg/mL (Figure 1A). Furthermore, 24 h old, established biofilms of Xen29 were also dose-dependently disrupted by farnesol, with 6 mg/mL of farnesol causing an over 50,000-fold reduction in colony-forming units (CFU) (Figure 1B). The remarkable antibiofilm effects of farnesol were further visualized by Live/Dead viability assays of Xen29 biofilms (Figure 1C,D), followed by quantitative analysis of the biofilm images using Photoshop^®^ (25.6.0 Release) for fluorescence intensity and Comstat2 (Version 2.1 1 July 2015) for analysis of the biomass and average thickness of biofilms (Figure S1). Farnesol was capable of both inhibition of biofilm formation and disruption of established biofilms by killing live (green) cells of *S. aureus* Xen29 in a dose-dependent fashion (Figure 1C,D and Figure S1). In the case of biofilm formation, the resulting dead (red) cells were scarcely attached to the surfaces (the weak red signal was only detectable by the Comstat2 software) (Figure S1D). However, for established biofilms, red fluorescence indicative of dead biofilm-encased cells peaked at 2 mg/mL of farnesol (Figure 1D and Figure S1C,E), suggesting that the dead cells were increasingly detached from the surfaces with increasing of the farnesol dose.

We also assessed the anti-biofilm activity of farnesol against *S. aureus* Xen40, a clinical strain and robust biofilm forming species. Farnesol was able to kill Xen40 cells in a dose-dependent manner and inhibit its biofilm formation, as indicated by the at least two-log CFU reduction with 1 mg/mL dose of farnesol (Figure S2A). This result was further confirmed by the Live/Dead viability assays of Xen40 developing biofilms and their corresponding quantitative analysis (Figure S2B–D). Moreover, farnesol killed over 99.9% (three-log CFU reduction) of biofilm-encased *S. aureus* Xen40 at 3 mg/mL of farnesol (Figure S3A). Surprisingly, the Live/Dead viability assays of Xen40 established biofilms shows that high doses (2–3 mg/mL) of farnesol caused apparent perforation and destruction of the established biofilms (Figure S3B), which is similar to the observation associated with the use of 0.2–0.5 mg/mL of farnesol on biofilm formation of Xen40 (Figure S2B). Interestingly, the dead (red) Xen40 cells killed by farnesol were still able to attach/stick to the surfaces so that the red fluorescence was much stronger compared to that of the Xen29 biofilms (compare Figure S2B to Figure 1C and Figure S3B to Figure 1D).

### 2.2. Farnesol Kills Persisters without Inducing Resistance

The antibiofilm effects of farnesol against *S. aureus* prompted us to further investigate its activity against *S. aureus* persister cells. We exposed 24 h old established biofilms of *S. aureus* Xen29 to a high dose of rifampicin [100× minimal inhibitory concentration (MIC)] for 24 h. Approximately 2.8% of the remaining viable biofilm-encased bacteria, called persister cells, survived the rifampicin treatment (Figure 1E). These persister cells were further exposed to the vehicle control, or different doses of farnesol for an additional 24 h. Farnesol killed the persister cells of Xen29 in a dose-dependent fashion, with 3 mg/mL of farnesol resulting in a 2000-fold reduction in CFU (Figure 1E), demonstrating that farnesol is bactericidal to not only planktonic and biofilm-encased cells, but also to tough-to-kill persister cells of *S. aureus* Xen29.

Since resistance development is a huge concern for conventional antibiotics, we also evaluated the ability of *S. aureus* Xen29 to develop resistance to farnesol. Serial passaging of Xen29 in the presence of subinhibitory (1/2× MIC) concentrations of farnesol did not select isolates resistant to farnesol, even after 20 continuous passages. In contrast, exposure to the antibiotic rifampicin triggered a rapid increase in MIC after just six passages, eventually resulting in a 16,384-fold increased MIC after 19 passages (Figure 1F). These results indicate that farnesol is capable of reducing persistence and resistance of *S. aureus* biofilms.

### 2.3. Farnesol Is Effective against P. aeruginosa Biofilms

**Figure 2:**
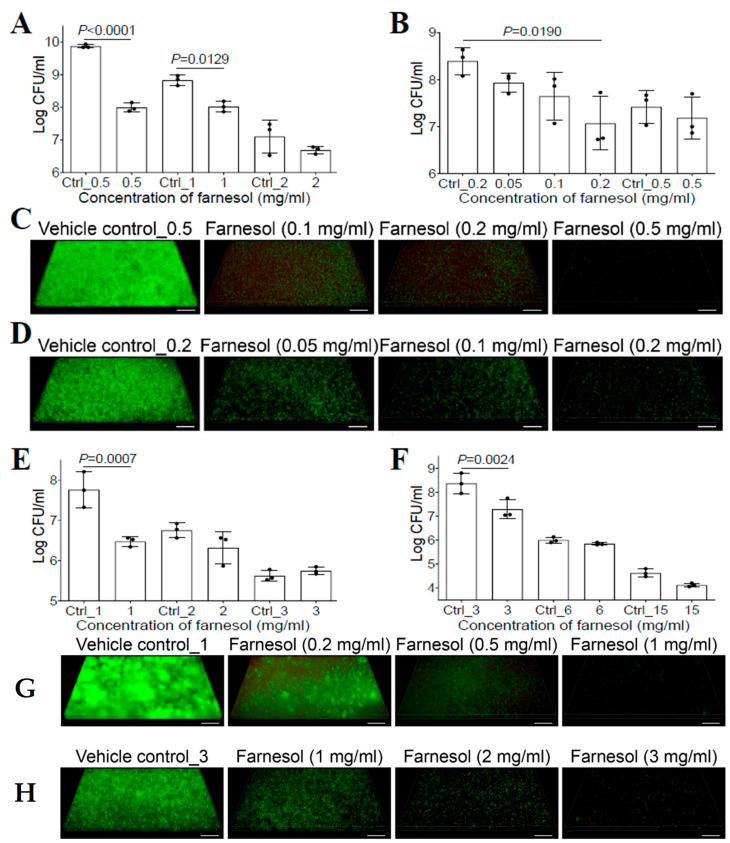
Farnesol inhibits biofilm formation and disrupts established biofilms of *P. aeruginosa*. (**A**) Inhibition of biofilm formation of *P. aeruginosa* Xen5 by farnesol 24 h after incubation in TSB in collagen-precoated wells. (**B**) Disruption of established biofilms of *P. aeruginosa* Xen5 followed by 24 h exposure to farnesol. (**C**,**D**) Three-dimensional merged images of Live/Dead viability of *P. aeruginosa* Xen5 biofilms after 24 h incubation (developing biofilm) (**C**) or 24 h exposure of 24 h old established biofilms (**D**) in TSB containing farnesol in collagen-precoated chambers. (**E**) Inhibition of biofilm formation of *P. aeruginosa* PAO1 by farnesol 24 h after incubation in TSB in collagen-precoated wells. (**F**) Disruption of established biofilms of *P. aeruginosa* PAO1 followed by 24 h exposure to farnesol. (**G**,**H**) Three-dimensional merged images of Live/Dead viability of *P. aeruginosa* PAO1 biofilms after 24 h incubation (**G**) or 24 h exposure of 24 h old established biofilms (**H**) in TSB containing farnesol in collagen-pre-coated chambers. The biofilms (**C**,**D**,**G**,**H**) were stained with both SYTO^®^ 9 (green fluorescence for live cells) and propidium iodide (red fluorescence for dead cells). Scale bars, 20 μm. Data in (**A**,**B**,**E**,**F**) are expressed as the number of viable bacteria in log10 CFU per mL and are shown as mean ± SD (n = 3). Ctrl_0.2 = 0.67% of ethanol; Ctrl_2 = 6.7% of ethanol; and Ctrl_15 = 50% of ethanol. See meanings of other Ctrl_# in Figure 1 legend.

Previous studies have shown that farnesol had no effect on the bacterial growth of *P. aeruginosa* for either the PAO1 [20] or PA14 strain [27]. However, the success of farnesol against *S. aureus* described above suggested that the new formulation of farnesol might be equally effective against other microbial biofilms. Thus, we reassessed the activity of farnesol against *P. aeruginosa* biofilms. We demonstrated that biofilm formation of *P. aeruginosa* Xen5 on collagen-coated plates was inhibited with 0.5 mg/mL of farnesol, causing an almost two-log reduction in CFU (Figure 2A). Moreover, 24 h old established biofilms of Xen5 were also significantly disrupted by farnesol in a dose-dependent manner, with 0.2 mg/mL of farnesol resulting in a more than 90% reduction in CFU (Figure 2B). The anti-biofilm effects of farnesol were visualized by the Live/Dead viability assays of Xen5 biofilms (Figure 2C,D), and confirmed by quantitative analysis of the biofilm images (Figure S4). Farnesol killed live (green) cells of *P. aeruginosa* Xen5 in a dose-dependent fashion, and the resulting dead (red) cells barely adhered to the surfaces (Figure 2C and Figure S4A). The killing of Xen5 cells was confirmed by the maximum weak (but detectable by Comstat2) red fluorescence at 0.1 mg/mL of farnesol, and the dead Xen5 cells failed to attach to the surfaces with the increased farnesol doses (Figure S4C). In established Xen5 biofilms, we observed the gradual decrease in live (green) biofilm-encased cells with increased farnesol doses; however, there was no corresponding increase in dead (red) biofilm-encased cells of Xen5 (Figure 2D and Figure S4B,D).

To rule out the possibility that the observed differences were due to the different *P. aeruginosa* strain used in a previous study [20], we assessed the activity of farnesol against *P. aeruginosa* PAO1. Similar to the effect against Xen5 biofilms, farnesol also killed PAO1 cells to inhibit its biofilm formation, as indicated by an approximately 95% reduction in CFU with 1 mg/mL of farnesol (Figure 2E). This result was further confirmed by the Live/Dead viability assays of PAO1-developing biofilms (Figure 2G), and their corresponding quantitative analyses (Figure S5A,C). Furthermore, farnesol also significantly disrupted 24 h old established biofilms of PAO1, with 3 mg/mL of farnesol causing over 90% reduction in CFU (Figure 2F). This result was further confirmed by the Live/Dead viability assays of PAO1-established biofilms (Figure 2H) and their corresponding quantitative analyses (Figure S5B,D). Interestingly, similar to the established Xen5 biofilms, we observed the same occurrences for established PAO1 biofilms; there was no evidence of dead (red) biofilm-encased PAO1 cells killed by farnesol, although farnesol dose-dependently reduced live (green) biofilm-encased PAO1 cells (Figure 2H and Figure S5B,D). The mechanism underlying this observation will be explored later. Of note, for all biofilm cases of Xen5 and PAO1 in vitro, the effects of higher doses of farnesol on *P. aeruginosa* biofilms were unexpectedly masked due to the toxic effects of the vehicle (ethanol) at higher concentrations (Figure 2A,B,E,F).

### 2.4. Farnesol Combats Bacteria by Direct Killing and Biofilm Detachment

**Figure 3:**
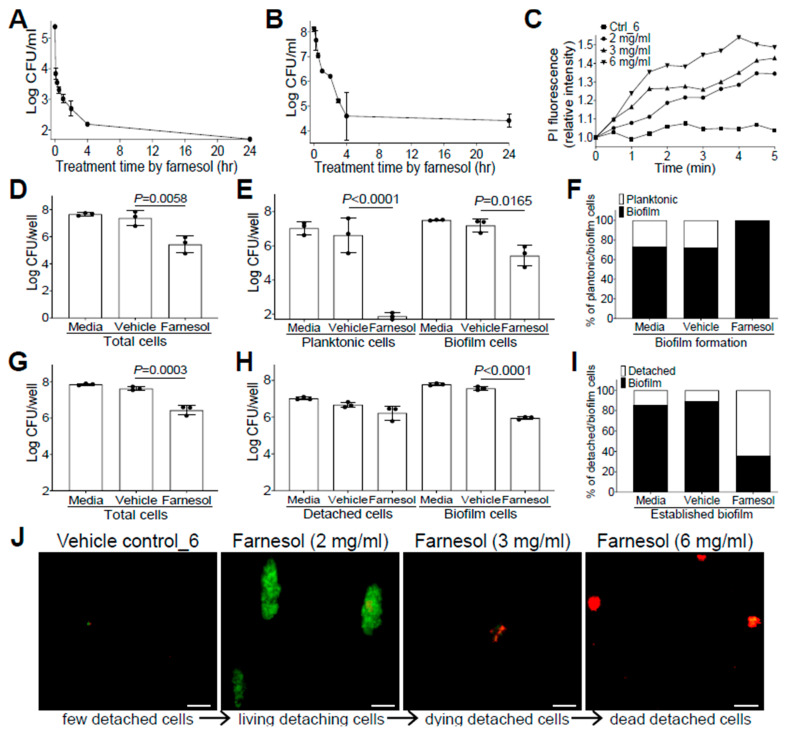
Farnesol combats *S. aureus* by direct killing and biofilm detachment. (**A**) Fast killing of planktonic cells of *S. aureus* Xen29 by farnesol (1 mg/mL) to eliminate its biofilm formation. (**B**) Disruption of 24 h old established biofilms of *S. aureus* Xen29 within four h of exposure to farnesol (6 mg/mL). (**C**) Killing of *S. aureus* Xen29 by farnesol as measured by propidium iodide (PI) influx. Data were normalized to the vehicle control at time zero and shown as the mean of three replicates. (**D**–**F**) Biofilm development: Vital cell counts of *S. aureus* Xen29 in total cells (**D**), planktonic and biofilm cells (**E**), and percentage of planktonic/biofilm cells in total cells (**F**) 24 h after incubation in NB1 containing 0.1 mg/mL of farnesol in plasma-precoated wells. (**G**–**I**) Established biofilms: Vital cell counts of *S. aureus* Xen29 in total cells (**G**), detached and biofilm cells (**H**), and percentage of detached/biofilm cells in total cells (**I**) after 24 h exposure of 24 h old established biofilms to 1 mg/mL of farnesol in plasma-pre-coated wells. Results in (**A**,**B**,**D**,**E**,**G**,**H**) are expressed as the number of viable bacteria in log_10_ CFU per mL. Data are shown as mean ± SD (n = 3). The *X*-axis line in (**A**,**E**) represents the lower limit of detection (Log 50 ≈ 1.7). (**J**) Biofilm detachment and killing of *S. aureus* Xen29 detached cells after 24 h exposure of 24 h old established biofilms to farnesol in plasma-pre-coated chambers as measured by Live/Dead viability assay. The detached cells were stained with both SYTO^®^ 9 (green fluorescence for live cells) and propidium iodide (red fluorescence for dead cells). Scale bars, 20 μm.

The anti-biofilm effects of farnesol against drug-resistant Gram-positive *S. aureus*, and Gram-negative *P. aeruginosa* impelled us to further explore its mechanisms as a potential broad-spectrum anti-biofilm agent. Since farnesol is known to induce the disruption of *S. aureus* membranes within min [28], we wanted to examine how rapidly farnesol kills planktonic cells of *S. aureus* Xen29 and disrupts its established biofilms. As expected, farnesol (1 mg/mL) killed about 97% of Xen29 planktonic cells quickly (within five min) and eliminated the bacteria completely after overnight incubation (Figure 3A). Furthermore, 6 mg/mL of farnesol quickly disrupted the 24 h old, established biofilms of Xen29, resulting in a 92% reduction in CFU for biofilm-encased cells within 30 min, and more than 99.9% reduction in CFU in four h (Figure 3B). The potential for farnesol to kill the bacteria was further confirmed by propidium iodide (PI) influx measurement. Farnesol initiated PI influx in increased proportion to both time and dose, whereas the vehicle control had no effect on the PI influx (Figure 3C). Similar PI influx results also occurred with *S. aureus* Xen40 and *P. aeruginosa* Xen5 and PAO1 strains, except that the vehicle controls for *P. aeruginosa* strains also caused a moderately increased PI influx, which is consistent with the vehicle killing of *P. aeruginosa* described above (Figure S6).

Farnesol has also been found to induce the detachment of established *Staphylococcus epidermidis* biofilms without cell killing [29]. To assess potential biofilm detachment of *S. aureus* Xen29 by farnesol, we selected an intermediate concentration of farnesol (0.1 mg/mL for prevention of biofilm formation; 1 mg/mL for treatment of established biofilm) to minimize the possible shielding effect of cell killing on biofilm detachment. Comparisons of the vital cell counts of planktonic (or detached cells in the case of established biofilms), biofilm, and total cells, as well as the percentage of planktonic (or detached) vs. biofilm cells in total cells with/without the farnesol treatment demonstrated that farnesol caused significant CFU reductions in planktonic, biofilm, and total cells, confirming the farnesol killing of Xen29 planktonic cells at doses as low as 0.1 mg/mL (Figure 3D,E). Moreover, farnesol killed over 99.999% of Xen29 planktonic cells during biofilm formation, so that almost 100% of the remaining vital cells were biofilm encased (Figure 3E,F). In established biofilms, 1 mg/mL of farnesol resulted in significant CFU reductions in both biofilm and total cells, but not detached cells of Xen29, indicating that, besides the capacity for killing biofilm-encased cells, lower doses of farnesol could also detach biofilm from the surface without cell killing (Figure 3G,H). The latter finding was further confirmed by the percentage of detached vs. biofilm cells, which showed that the percentage of viable detached cells had increased more than five-fold (Figure 3I). Furthermore, we visualized biofilm detachment and killing of Xen29 cells by farnesol using the Live/Dead viability assay. The lower dose (2 mg/mL) of farnesol triggered the detachment of biofilm from the established biofilms without cell killing (as indicated by green fluorescence). However, the higher doses (3 or 6 mg/mL) of farnesol ultimately killed the detached cells (red fluorescence) (Figure 3J).

**Figure 4:**
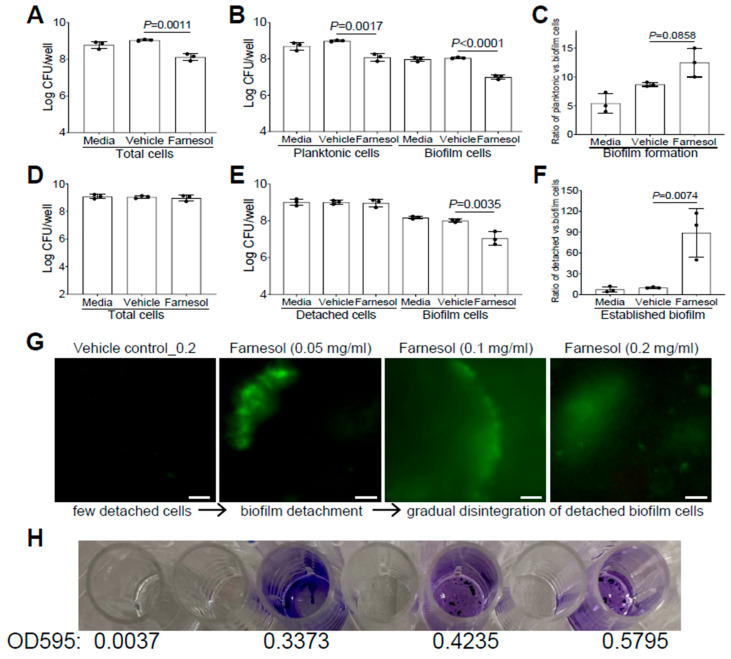
Farnesol kills planktonic cells of *P. aeruginosa* but disrupts its established biofilms mainly by biofilm detachment in vitro. (**A**–**C**) Vital cell counts of *P. aeruginosa* Xen5 in total cells (**A**), planktonic and biofilm cells (**B**), and ratios of planktonic vs. biofilm cells (**C**) 24 h after incubation in TSB (developing biofilm) containing 0.5 mg/mL of farnesol in collagen-precoated wells. (**D**–**F**) Vital cell counts of *P. aeruginosa* Xen5 in total cells (**D**), detached and biofilm cells (**E**), and ratios of detached vs. biofilm cells (**F**) after 24 h exposure of 24 h old established biofilms to 0.2 mg/mL of farnesol in collagen-precoated wells. Results in (**A**,**B**,**D**,**E**) are expressed as the number of viable bacteria in log_10_ CFU per mL. Data are shown as mean ± SD (n = 3). (**G**,**H**) Biofilm detachment and disintegration of *P. aeruginosa* Xen5 detached cells after 24 h exposure of 24 h old established biofilms to farnesol in collagen-precoated chambers as measured by Live/Dead viability assay (**G**) and crystal violet staining (**H**); the top labels in (**G**) are shared in (**H**). The detached cells (**G**) were stained with both SYTO^®^ 9 (green fluorescence for live cells) and propidium iodide (red fluorescence for dead cells). Scale bars, 20 μm.

Similarly, we also evaluated whether farnesol uses the same mechanisms to fight against *P. aeruginosa.* Farnesol was found to kill Xen5 planktonic cells, as demonstrated by significant CFU reductions in planktonic, biofilm, and total cells, compared with the vehicle controls (Figure 4A,B). However, there were no significant increases (*p* = 0.0858) in the ratios of planktonic vs. biofilm cells for biofilm formation of Xen5 by farnesol (Figure 4C), suggesting that planktonic cell killing by farnesol is the dominant mechanism for the inhibition of *P. aeruginosa* biofilm formation in vitro. In established biofilms of Xen5, farnesol only caused significant CFU reductions of biofilm-encased cells, but not for either the detached or total cells (Figure 4D,E). This implies that farnesol might detach established biofilms rather than killing biofilm-encased cells at the farnesol dose employed (0.2 mg/mL). This was confirmed by the nine-fold increase in the average ratios of detached vs. biofilm cells by farnesol (Figure 4F). Moreover, we visualized the detachment and disintegration of Xen5 established biofilms by farnesol using the Live/Dead viability assay and crystal violet staining. Consistent with the above CFU data for biofilm detachment, lower doses (0.05 mg/mL) of farnesol-initiated biofilm detachment in the established Xen5 biofilms formed detached cells in the supernatant, while higher doses (0.1–0.2 mg/mL) of farnesol seemed to gradually break up the detached cells without cell killing (retaining green fluorescence) (Figure 4G). Consistent with the Live/Dead viability assay, crystal violet staining of the detached cells treated at the range of 0.05 to 0.2 mg/mL of farnesol showed a gradual disintegration of the biofilm (Figure 4H). In addition, we did not observe any potential killing effects of the vehicle, since there were essentially no differences between the vehicle controls and media controls (Figure 3D–I and Figure 4A–F).

### 2.5. Farnesol Is Effective against Polymicrobial Biofilms

**Figure 5:**
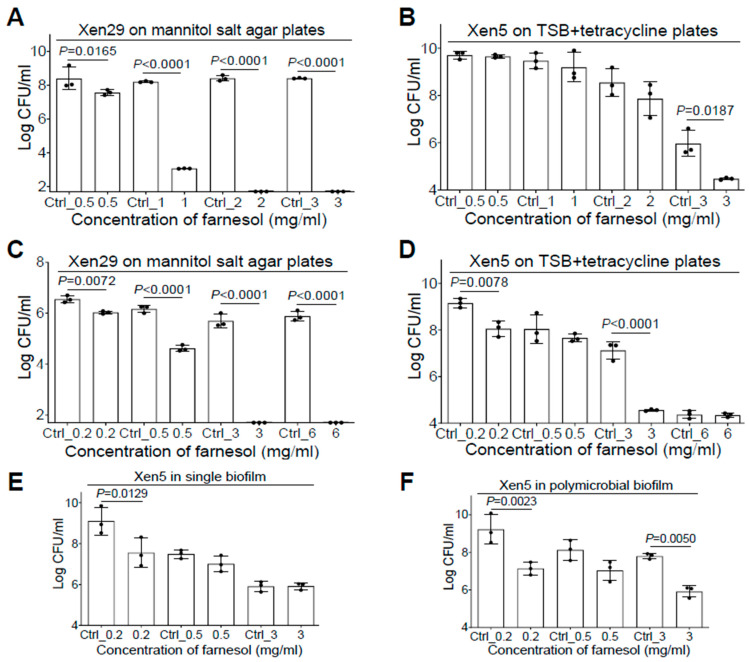
Farnesol inhibits mixed biofilm formation and disrupts established polymicrobial biofilms of both *P. aeruginosa* and *S. aureus*. (**A**,**B**) Inhibition of mixed biofilm formation of both *S. aureus* Xen29 (**A**) and *P. aeruginosa* Xen5 (**B**) by farnesol 24 h after incubation in TSB containing 5% BSA in collagen-precoated wells. (**C**,**D**) Disruption of established polymicrobial biofilms of both *S. aureus* Xen29 (**C**) and *P. aeruginosa* Xen5 (**D**) followed by 24 h exposure to farnesol. The *X*-axis line in (**A**,**C**) represents the lower limit of detection (Log 50 ≈ 1.7). (**E**,**F**) Side-by-side comparison for disruption of established biofilms of *P. aeruginosa* Xen5 by farnesol in single (**E**), or polymicrobial biofilms with *S. aureus* Xen29 (**F**). Mannitol salt agar (**A**,**C**) was used to select for Xen29, whereas tetracycline (100 µg/mL) (**B**,**D**,**F**) was used to select for Xen5, from the polymicrobial biofilms. Data are expressed as the number of viable bacteria in log10 CFU per mL and are shown as mean ± SD (n = 3). See meanings of Ctrl_# in legends of Figure 1 and Figure 2.

Since co-occurrence of *S. aureus* and *P. aeruginosa* in vivo has been linked to poor progress of biofilm-related infections such as cystic fibrosis and chronic/burn wounds [8,30], we sought to investigate the anti-biofilm effect of farnesol against *S. aureus*—*P. aeruginosa* polymicrobial biofilms. With the addition of 5% of bovine serum albumin (BSA) in TSB [31], we were able to successfully generate the Xen29-Xen5 polymicrobial biofilms in vitro. Interestingly, both species of bacteria seemed to protect one another from the effects of farnesol in the mixed biofilm formation. For example, Xen29 could survive cultures in 1 mg/mL of farnesol in the presence of Xen5 (compare Figure 5A with Figure 1A). Xen29 appears to protect Xen5 from both farnesol killing at 0.5 mg/mL and vehicle killing at high doses (Ctrl_1 = 3.3% of ethanol; Ctrl_2 = 6.7% of ethanol) in the mixed biofilms (compare Figure 5B with Figure 2A). In established polymicrobial biofilms, the interaction between Xen29 and Xen5 produced complicated outcomes. Xen29 seemed to be more sensitive to farnesol killing since 3 mg/mL of farnesol was enough to eradicate it in the established polymicrobial biofilms (compare Figure 5C with Figure 1B). In contrast, Xen29 seemed to protect Xen5 from the vehicle killing at Ctrl_3 (=10% of ethanol), so that 3 mg/mL of farnesol caused a 400-fold reduction in CFU of Xen5 compared to the vehicle control in the established polymicrobial biofilms (Figure 5D). To rule out the possibility that the Xen5 survival from vehicle killing might be due to the addition of 5% BSA in the co-culture, we performed a side-by-side comparison to evaluate disruption of Xen5 established biofilms by farnesol in single vs. polymicrobial biofilms with Xen29 in the TSB + 5% BSA media. We repeated the farnesol anti-biofilm activity at 0.2 mg/mL against Xen5 established biofilms (compare Figure 5E with Figure 2B), indicating that the BSA addition had no effect on the farnesol’s anti-biofilm activity. Furthermore, Xen5 could survive the vehicle (Ctrl_3) killing only in the presence of Xen29 (Figure 5F), demonstrating that Xen29 protects Xen5 from vehicle killing in polymicrobial biofilms. This demonstrates that, besides its significant activity against single biofilms of either *S. aureus* or *P. aeruginosa*, farnesol is also effective for inhibiting mixed biofilm formation and disrupting established polymicrobial biofilms containing both *S. aureus* and *P. aeruginosa*.

### 2.6. Farnesol Is Effective and Safe against Biofilm-Related Skin Infections

**Figure 6:**
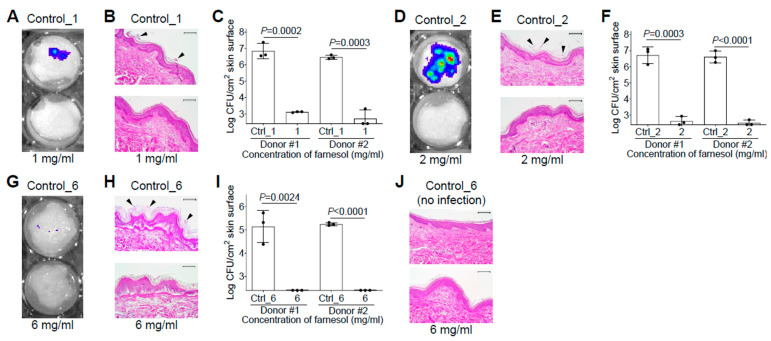
Farnesol is highly effective and safe for both the prevention and treatment of biofilm-associated infections of *S. aureus* on ex vivo intact or burned human skin. (**A**–**C**) Prevention of biofilm-associated infections of *S. aureus* Xen29 by farnesol (1 mg/mL) 48 h after inoculation on ex vivo intact human skin, as assayed by luminescence measurement using the in vivo imaging system (IVIS) (**A**), light micrographs of hematoxylin and eosin (H&E)-stained crossing sections of skin biopsies (**B**), and vital cell counts (**C**). (**D**–**F**) Proximate elimination of established biofilm infections of *S. aureus* Xen29 after 24 h inoculation followed by 24 h exposure to farnesol on ex vivo intact human skin, as assayed by luminescence measurement using IVIS (**D**), light micrographs of H&E-stained crossing sections of skin biopsies (**E**), and vital cell counts (**F**). (**G**–**I**) Eradication of established biofilm infections of *S. aureus* Xen29 after 24 h inoculation followed by 24 h exposure to farnesol on ex vivo burned human skin, as assayed by luminescence measurement using IVIS (**G**), light micrographs of H&E-stained cross-sections of skin biopsies (**H**) and vital cell counts (**I**). (**J**) Safety of topical application of farnesol on human skin. Intact human skin was treated for 48 h with 6 mg/mL of farnesol, or its corresponding vehicle control. Results are shown as the light micrographs of H&E-stained cross-sections of skin biopsies. Arrowheads in (**B**,**E**,**H**) indicate biofilm formation or establishment on skin. Data in (**C**,**F**,**I**) are expressed as the number of viable bacteria in log10 CFU per square centimeter (cm^2^) of skin surface and are shown as mean ± SD (n = 3). The *X*-axis line in (**C**,**F**,**I**) represents the lower limit of detection (Log 255 ≈ 2.4). Scale bars, 100 μm. See meanings of Ctrl_# in legends of Figure 1 and Figure 2.

Since *S. aureus* and *P. aeruginosa* are among the most common pathogens responsible for infections in burn patients [32], we would like to examine the efficiency of farnesol against biofilms developed on skin using ex vivo intact, or burned, human skin. Skin was first inoculated with *S. aureus* Xen29 in the presence and absence of 1 mg/mL of farnesol for 48 h. Consistent with the in vitro data, farnesol prevented Xen29 biofilm formation on intact human skin, as visualized by a significant decrease in luminescence (Figure 6A), and the disappearance of Xen29 biofilm on the top of epidermis (Figure 6B). This result was further confirmed by an over 5000-fold CFU reduction of Xen29 on the skin (Figure 6C). For an unknown reason, Xen29 failed to form biofilm on the burned skin even for the positive (vehicle) control; hence, additional study of Xen29 biofilm formation on ex vivo burned skin was not pursued. Farnesol (2 mg/mL) almost eliminated 24 h old, established biofilms of Xen29 on intact human skin, as demonstrated by the dramatically reduced luminescence (Figure 6D), vanishing of biofilms on the skin surface (Figure 6E), and a more than 4-log (>12,000-fold) reduction in CFUs (Figure 6F). Although *S. aureus* Xen29 seemed to grow slowly (compare Figure 6I with Figure 6F) and produce less luminescence (compare Figure 6G with Figure 6D) on burned human skin compared to intact human skin, it still established biofilms on the burned epidermis, which was ruptured due to the burn wound creation (Figure 6H). Remarkably, 6 mg/mL of farnesol was sufficient to eradicate the established biofilm infections of Xen29 on the burned skin (Figure 6I). In consideration of safety, farnesol concentrations as high as 6 mg/mL had no observable side effects on the ex vivo human skin over the 48 h culture period (Figure 6J), consistent with the findings of a previous review that up to 12% (=120 mg/mL) farnesol is safe to apply to human skin as a fragrance ingredient [10]. These results demonstrate that farnesol is highly effective, and safe, for both the prevention and treatment of biofilm-associated infections of *S. aureus* ex vivo.

**Figure 7:**
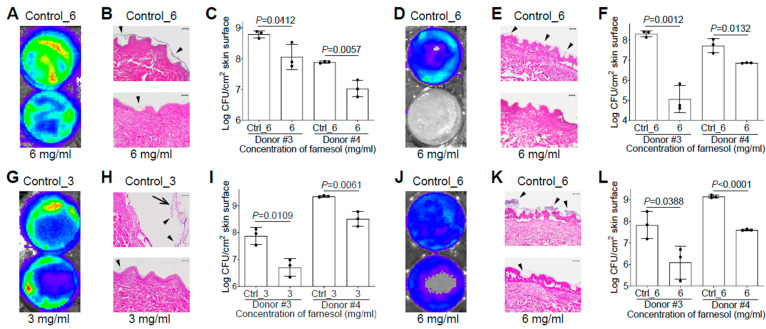
Farnesol is effective for both the prevention and treatment of biofilm-associated infections of *P. aeruginosa* in ex vivo intact or burned human skin. (**A**–**C**) Inhibition of biofilm-associated infections of *P. aeruginosa* Xen5 by farnesol (6 mg/mL) 24 h after inoculation on ex vivo intact human skins as assayed by luminescence measurement using IVIS (**A**), light micrographs of H&E-stained cross sections of skin biopsies (**B**) and vital cell counts (**C**). (**D**–**F**) Inhibition of biofilm-associated infections of *P. aeruginosa* Xen5 by farnesol (6 mg/mL) 24 h after inoculation on ex vivo burned human skin as assayed by luminescence measurement using IVIS (**D**), light micrographs of H&E-stained cross sections of skin biopsies (**E**) and vital cell counts (**F**). (**G**–**I**) Alleviation of established biofilm infections of *P. aeruginosa* Xen5 after 24 h inoculation followed by 24 h exposure to farnesol on ex vivo intact human skins as assayed by luminescence measurement using IVIS (**G**), light micrographs of H&E-stained crossing sections of skin biopsies (**H**), and vital cell counts (**I**). (**J**–**L**) Mitigation of established biofilm infections of *P. aeruginosa* Xen5 after 24 h inoculation followed by 24 h exposure to farnesol on ex vivo burned human skin, as assayed by luminescence measurement using IVIS (**J**), light micrographs of H&E-stained cross sections of skin biopsies (**K**), and vital cell counts (**L**). Arrows in (**H**) indicate peeled epidermis, which had to be artificially replaced for H&E staining; arrowheads in (**B**,**E**,**H**,**K**) indicate biofilm formation or establishment on skin. Data in (**C**,**F**,**I**,**L**) are expressed as the number of viable bacteria in log10 CFU/cm^2^ of skin surface and are shown as mean ± SD (n = 3). Scale bars, 100 μm. See meanings of Ctrl_# in Figure 1 legend.

We also evaluated the efficacy of farnesol against biofilm-associated skin infections of *P. aeruginosa* Xen5 using the ex vivo intact or burned human skin model. It surprised us that there was no vehicle (ethanol) killing of Xen5 on the ex vivo human skin, allowing 6 mg/mL of farnesol to be used against the Xen5 skin infection. Compared to the vehicle control, farnesol (6 mg/mL) significantly inhibited Xen5 biofilm formation on intact human skin, as indicated by reduced luminescence intensity (Figure 7A), diminished biofilm on the epidermis (Figure 7B), and substantial CFU reductions for two independent human samples (Figure 7C). Similar results occurred for preventing Xen5 biofilm formation on the burned human skin by the farnesol treatment (Figure 7D–F). Damage caused by the established Xen5 biofilm infection on human skin was so severe so that the epidermis turned green and could be easily removed; however, 3 mg/mL of farnesol provided protection from the severe necrosis and epidermal disruption (Figure S7). This farnesol protection against the Xen5 infection on intact skin was further confirmed by in vivo imaging system (IVIS) imaging (Figure 7G), and hematoxylin and eosin (H&E) staining of the cross-sectioned skin biopsies (Figure 7H), plus significant CFU reductions (Figure 7I). Furthermore, farnesol (6 mg/mL) is also effective against the Xen5 infection on burned skin, as demonstrated by the decreased intensity of luminescence (Figure 7J), and biofilm reduction on the epidermis (Figure 7K), as well as a significant decrease in CFUs (Figure 7L). It is worth mentioning that the farnesol treatment against the 24 h old, established Xen5 biofilm infections on intact/burned skin was administrated at the center area of the skin samples, which is the reason why the central regions show the best protection of farnesol compared to the edges (Figure 7G,J).

### 2.7. Farnesol Is Not Toxic and Protects HEKa from Ethanol Killing In Vitro

**Figure 8:**
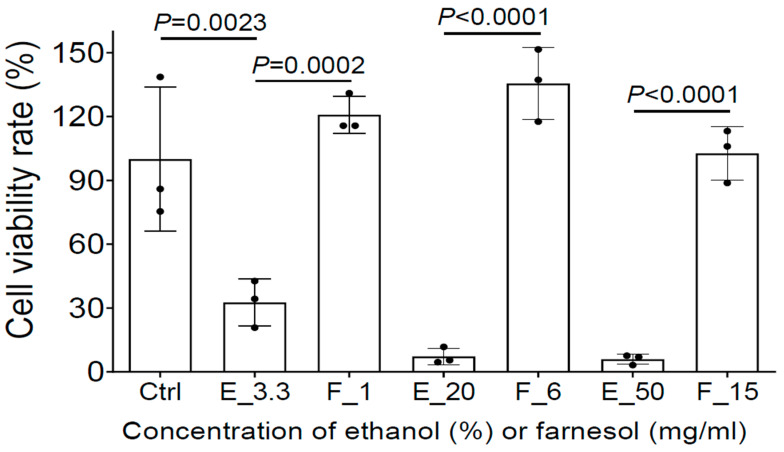
Farnesol is not toxic and further protects HEKa from apparent ethanol killing. The potential cytotoxicity of three farnesol doses (1, 6, 15 mg/mL) and their corresponding vehicle (ethanol) controls on HEKa monolayers was evaluated by MTS assay. Ctrl, Media control; E_# represents the ethanol percentage (%) in the vehicle control; F_# represents the farnesol concentrations in the same amount of ethanol as the ethanol controls, in the units mg/mL.

To further evaluate whether farnesol is safe for the treatment of open-wound skin infections, MTS assay was used to assess the potential cytotoxicity of farnesol/ethanol on human epidermal keratinocytes from adults (HEKa), the most dominant cells in the epidermal layer of skin playing a critical role in wound healing [33]. Although as low as 3.3% ethanol reduced cell viability to HEKa, farnesol was not toxic to HEKa, and appears to offer a protective benefit against ethanol toxicity. Among the three farnesol doses (1, 6, or 15 mg/mL), 6 mg/mL of farnesol showed the best outcome, although all of three doses are safe to HEKa (Figure 8). Collectively, these results strongly suggest that farnesol is safe for both the prevention and treatment of intact or open-wound skin infections.

## 3. Discussion

Farnesol is oily by nature, making it difficult to disperse in aqueous media, hence we have used it as an emulsion by first dissolving it in ethanol. Here we report that farnesol, a natural product commercially available as a cosmetic and flavor enhancer, is highly effective for preventing biofilm formation, and can also disrupt established biofilms of Gram-positive *S. aureus* or Gram-negative *P. aeruginosa*, two notorious members of the ESKAPE pathogen family [34], both in vitro and ex vivo. Our results indicate that farnesol is bactericidal by cell membrane permeabilization. Ethanol alone can kill bacteria, but we have shown that there is a profound effect of farnesol compared to ethanol when used against *S. aureus*. We also show that farnesol is effective against *P. aeruginosa*, although not as effective as against *S. aureus*. Furthermore, farnesol is effective against both species of bacteria in a polymicrobial biofilm, which is most common in burn wounds. Interestingly, farnesol has a greater impact against *P. aeruginosa* in a polymicrobial biofilm than against single species, which was an unexpected resulting, necessitating further exploration. Besides killing planktonic and biofilm-encased cells, farnesol is also able to kill *S. aureus* persister cells, which are the dormant and drug-tolerant subpopulation. We demonstrated a lack of resistance development to farnesol by *S. aureus*, even after prolonged culture in the presence of sub-inhibitory farnesol doses. This suggests that farnesol may be superior to classical antibiotics since *S. aureus* remained sensitive to its antimicrobial effects.

A key feature of farnesol is its ability to induce biofilm detachment, and both halts biofilm development and stimulates the release of mature biofilm from a surface. Wound care for burn wounds and chronically infected wounds, such as diabetic foot ulcers, requires weekly debridement to remove biofilm before the application of a subsequent redressing; however, debridement is often not entirely effective. We propose that farnesol could be incorporated into debridement irrigation solutions for enhanced removal of biofilm from the wound bed. We have also demonstrated that farnesol is capable of detaching biofilm from surfaces without cell killing. This gives farnesol an extra advantage by removing the biofilms from surfaces/tissues while minimizing its potential side effects on the host or environment. Mechanisms underlying this biofilm detachment are not yet clear, although they most likely stem from the hydrophobic nature of farnesol in emulsion form which can serve as a disruptive interface between biofilm and a surface, or between bacteria within biofilm. Farnesol has been found to dissolve fibrin fibers of established biofilms of *S. aureus* [35]. Since the fibers seem to play important roles in connecting the biofilm-encased cells together, their dissolution by farnesol could contribute to the potential mechanism for biofilm detachment. Since farnesol is able to kill both Gram-positive and negative bacteria quickly, it is likely that its bactericidal activity disrupts bacterial membranes from the outside (including persister cells). Furthermore, farnesol is also able to detach and disintegrate biofilms, exposing hidden persister cells protected by the biofilm matrix to allow cell killing.

Farnesol is FDA approved for topical application (up to 120 mg/mL) as a fragrance enhancer on human skin [10]; however, its repurposing as a potential antimicrobial has not been extensively examined for impeding *S. aureus* and *P. aeruginosa*, and their polymicrobial biofilms in wounds. *P. aeruginosa* and *S. aureus* often engage in a symbiotic relationship in wounds, and the use of one agent to eliminate them both would be beneficial. Farnesol is more effective against *S. aureus* than *P. aeruginosa*; nonetheless, its potential to halt *P. aeruginosa* biofilm formation and facilitate detachment is novel. Current antibiotics are notoriously ineffective against biofilm, and do not often induce biofilm detachment and disintegration, which appear to be key methods by which farnesol is effective. Notably, there is enhanced benefit of farnesol against *P. aeruginosa* biofilm on skin (Figure 7) compared to on plastic (Figure 2), hinting that there may be unknown mechanisms by which farnesol on skin is effective. Plus, farnesol seems to be more effective on burned skin compared to intact skin.

We also confirmed that 6 mg/mL of farnesol in 20% ethanol (a five-fold dilution with media from 30 mg/mL of stock) was safe without any adverse signs of skin damage in an ex vivo human skin model. Moreover, we demonstrated that up to 15 mg/mL of farnesol is not only safe to keratinocytes, but also protects HEKa cells from apparent ethanol killing in vitro. Our results suggest that topical application of farnesol emulsion (e.g., topically, or incorporated in wound dressings) holds promise as an affordable and effective treatment for a variety of clinical challenges, including burn wounds. Furthermore, due to its versatile activities against Gram negative *P. aeruginosa*, and Gram-positive *S. aureus*, as we have shown here, it is also possible that farnesol could be effective against a wide variety of microbes, significantly expanding its current therapeutic targets and applications.

In brief, we have demonstrated that farnesol emulsion is a prospective candidate to combat challenging biofilm infections due to its excellent anti-biofilm activity and its superiority in reducing persistent and resistant bacterial populations in biofilms of *S. aureus* and *P. aeruginosa,* including their polymicrobial biofilms. Moreover, its proven safety and extremely low cost makes farnesol a potential replacement to the prevalent disinfectants or antiseptics (e.g., 70% ethanol, bleach, and iodine tincture) used in current hospital settings.

## 4. Materials and Methods

### 4.1. Bacterial Strains and Culture

The bacterial strains used in this study were *S. aureus* (ATCC 12600, the strain of this species, and UAMS-1) and *P. aeruginosa* (ATCC 19,660 and PAO1). The first three strains were engineered for bioluminescence and designated *S. aureus* Xen29, Xen40, and *P. aeruginosa* Xen5, respectively (Perkin Elmer, Waltham, MA, USA) [36,37]. For single bacterial cultures, nutrient broth No 1 (NB1, Sigma, St. Louis, MO, USA) medium/agar was used to culture *S. aureus*, whereas tryptic soy broth without dextrose (TSB, Becton Dickinson, Franklin Lakes, NJ, USA) medium/agar was used to culture *P. aeruginosa*. Before each experiment, inoculum from a frozen stock was grown overnight on an NB1 or TSB plate at 37 °C. An isolated colony was then used to inoculate a fresh culture. Bacteria were cultured overnight at 37 °C and 160 rpm, and then centrifuged at 2000× *g* for 10 min. The pellet was resuspended in fresh broth, followed by the measurement of optical density at 600 nm (OD600). The concentration of CFU/mL was calculated based on a conversion formula correlating OD600 to CFUs (Figure S8, see below for details). The bacteria were then diluted to the desired concentrations (1 × 10^6^ CFU/mL for biofilm formation, or 1 × 10^8^ CFU/mL for established biofilm, unless otherwise stated). For CFU counting, samples were serially diluted, and drop-plated on NB1 or TSB agar for *S. aureus* and *P. aeruginosa*, respectively [38]. For polymicrobial culture, both Xen29 and Xen5 were grown in TSB containing 5% bovine serum albumin (BSA, Sigma) to facilitate *S. aureus/P. aeruginosa* co-existence [31]. Mannitol salt agar (Becton Dickinson) was used to select for *S. aureus,* while TSB agar containing tetracycline (100 µg/mL, Sigma) was used to select *P. aeruginosa* from the polymicrobial culture.

### 4.2. Conversion between OD600 and CFU/mL

To obtain the formula for conversion between OD600 and CFU/mL, a bacterial pellet was re-suspended in fresh broth and its OD600 was measured. The bacterial culture was then serially diluted to obtain OD600 readings of ~0.1, 0.2, 0.4, and 0.8. The CFU/mL of viable bacteria for each OD600 value was determined by serial dilutions and drop plating. The linear relationship for conversion between OD600 and CFU/mL for each strain, and their individual coefficient of determination (R^2^) values were analyzed using GraphPad Prism 9 (version 9.2.0).

### 4.3. Inhibition of Biofilm Formation

Biofilms were started from the overnight bacterial cultures as described above. Polystyrene plates (96-well) were coated with 250 µL of 20% filtered, apheresis-derived pooled human plasma (Innovative Research, Novi, MI, USA) in 50 mM of sodium bicarbonate (Sigma) for 24 h at 4 °C for *S. aureus.* Alternatively, plates were coated with 35 µg/mL of rat tail collagen type 1 (Corning, New York, NY, USA) in 0.02N acetic acid (Fisher, Waltham, MA, USA) for 1 h at room temperature for *P. aeruginosa,* or the polymicrobial culture. In the coated wells, bacteria (1 × 10^6^ CFU/mL) were cultured in 100 µL of NB1/TSB containing farnesol (Cayman Chemical, Ann Arbor, MI, USA, prepared as a 30 mg/mL of stock in ethanol, stored at −20 °C, diluted to various final concentrations with medium at the time of inoculation). For mixed biofilm formation, both Xen29 and Xen5 (1 × 10^5^ CFU/species/well) were mixed with farnesol in 100 µL of TSB containing 5% BSA. As a vehicle control, bacteria were exposed to the medium containing the same amount of ethanol but without farnesol. After 24 h incubation at 37 °C in a humidified container, planktonic cells were removed, biofilms were washed with 100 µL of phosphate-buffered saline (PBS), adherent bacteria were dislodged in 100 µL of PBS by vigorous (≥10 times) pipetting, and the CFU/mL of viable bacteria was determined by serial dilutions and drop plating. The lower limit of detection was 50 CFU/mL. To visualize the data on a logarithmic scale, a value of 50 CFU/mL was assigned when no growth occurred.

### 4.4. Treatment of Established Biofilms

Single biofilms were established by culturing 250 µL of NB1/TSB containing innocula (1 × 10^8^ CFU/mL) in a plasma/collagen-pre-coated 96-well plate at 37 °C. For established polymicrobial biofilms, 250 µL of mixed Xen29 and Xen5 innocula (1 × 10^7^ CFU/species/well) was cultured in TSB containing 5% BSA. After a 24 h incubation in a humidified container, planktonic cells were removed, and the established biofilms were washed with 250 µL of PBS, and then exposed to 100 µL of various final concentrations of farnesol in media. After an additional 24 h incubation in a humidified container, floating cells were removed, adherent biofilms were washed with 100 µL of PBS, and dislodged in 100 µL of PBS by vigorous (≥10 times) pipetting, and the CFU/mL of viable bacteria was determined by serial dilutions and drop plating. Biofilms were exposed to the same amount of ethanol in media as a vehicle control. The lower limit of detection was 50 CFU/mL. To visualize the data on a logarithmic scale, a value of 50 CFU/mL was assigned when no growth occurred.

### 4.5. Live/Dead Viability Assay

The effect of farnesol on biofilm formation and established biofilms of *S. aureus* or *P. aeruginosa* was visualized by FilmTracer™ (Invitrogen, Waltham, MA, USA) Live/Dead biofilm viability assay. Biofilms were established by culturing 500 µL of NB1/TSB containing innocula (1 × 10^6^ CFU/mL plus farnesol for biofilm formation; 1 × 10^8^ CFU/mL for established biofilm) in chambers of a 4-well Lab-Tek™ chambered coverglass (Nunc, Waltham, MA, USA) precoated with 500 µL of human plasma or rat tail collagen as described above. After 24 h incubation in a humidified container, planktonic cells were removed, and biofilms were washed with 500 µL of sterile water (for biofilm formation) or PBS (for established biofilms). The established biofilms were then exposed to 500 µL of farnesol in medium for an additional 24 h in a humidified container, then detached cells were removed, and adherent biofilms were further washed with 500 µL of sterile water. Established biofilms were stained for 20–30 min at room temperature with 250 µL of a mixture containing 10 µM of SYTO^®^ 9 green fluorescent nucleic acid stain and 60 µM of propidium iodide (PI) red-fluorescent nucleic acid stain, while protected from light. The biofilms were then washed with 250 μL of sterile water, covered with 300 μL of sterile water, and observed using a Keyence^®^ BZ-X800/BZ-X810 All-in-One fluorescence microscope (Keyence, Itasca, IL, USA). The obtained biofilm images were analyzed using Photoshop^®^ to quantify fluorescence intensity, and Comstat2 to evaluate three-dimensional biofilm structure [39,40].

For some experiments, following farnesol treatment, the supernatant containing detached cells (400 µL) was transferred into a sterile tube, centrifuged at 17,000× *g* for 5 min, then the supernatant was removed, and the pellet was washed with 1 mL of sterile water. The washed pellet was then re-suspended and stained with the 250 µL of SYTO^®^ 9-PI mixtures described as above, and spun down to remove unbound fluorescent dyes, washed with 1 mL of sterile water, re-suspended in 300 µL of sterilized water, and transferred into another pre-coated chambered coverglass. The stained cells were allowed to settle at room temperature for 30 min while protected from light, and then were imaged using the Keyence^®^ microscope.

In some cases, the washed pellet from 200 µL of detached cells was stained with 100 μL of 0.06% crystal violet in water for 10 min, then centrifuged to remove the unbound stain, washed twice with 1 mL of sterile water, then re-suspended in 100 µL of sterilized water and transferred into a 48-well plate. The purple detached cells were then photographed, followed by measurement of the optical density at 595 nm using a FilterMax F5 multi-mode microplate reader (Molecular Devices, San Jose, CA, USA).

### 4.6. Bactericidal Activity against Persister Cells

To obtain persister cells, biofilms were established by culturing 250 µL of NB1 containing *S. aureus* Xen29 innocula (1 × 10^8^ CFU/mL) in a plasma-pre-coated 96-well plate at 37 °C in a humidified container for 24 h. Planktonic cells were removed by washing with 250 µL of PBS, and biofilms were exposed to 100 µL of NB1 containing rifampicin (Bedford Laboratories, Bedford, OH, USA) (0.8 µg/mL = 100 × MIC). After 24 h incubation at 37 °C in a humidified container, media were removed followed by washing with 100 µL of PBS, and adherent bacteria containing persister cells were further exposed to 100 µL of various concentrations of farnesol in NB1. After an additional 24 h incubation in a humidified container, media were removed, and adherent biofilms were washed with 100 µL of PBS, then dislodged in 100 µL of PBS by vigorous (≥10 times) pipetting, and the CFU/mL of viable bacteria was determined by serial dilutions and drop plating. As a control, biofilms were exposed to same amount of vehicle in NB1.

### 4.7. Resistance Development

For comparison, development of resistance to the clinically relevant antibiotic rifampicin (Bedford Laboratories) was tested as a positive control. Five microliters of fresh *S. aureus* Xen29 inoculum (1 × 10^8^ CFU/mL) was combined with 95 µL of NB1 containing serial dilutions of farnesol (with final concentrations ranging from 0.008 to 1.024 mg/mL), or rifampicin (with final concentrations ranging from 0.004 to 0.512 µg/mL). Plates were double sealed with parafilm and incubated overnight at 37 °C and 160 rpm. The MIC, the lowest concentration of farnesol/rifampicin that caused lack of visible bacterial growth, was determined. Thereafter, the 0.5-fold MIC suspension was diluted five-fold with fresh NB1, and 5 µL of the diluent was added to 95 µL of fresh NB1 containing serial dilutions of farnesol/rifampicin, and these mixtures were incubated as described above. The ranges of farnesol or rifampicin concentrations were increased, if needed, based on the daily-updated MIC results. This was repeated for 20 continuous passages.

### 4.8. Time-Kill Assay

For biofilm formation, a fresh *S. aureus* Xen29 inoculum (1 × 10^6^ CFU/mL) was cultured in 100 µL of NB1 containing 1 mg/mL of farnesol in a plasma-pre-coated 96-well plate in a humidified container. After incubation at 37 °C for 0, 5, 15, and 30 min, or 1, 2, 4, and 24 h, bacteria in each well were quickly suspended by vigorous (≥10 times) pipetting, and the CFU/mL of viable bacteria was determined by serial dilution and drop plating. The vehicle control (no farnesol addition) was only added to the zero time point since farnesol seemed to kill Xen29 within a few seconds in the preliminary data. The lower limit of detection was 50 CFU/mL. To visualize the data on a logarithmic scale, a value of 50 CFU/mL was assigned when no growth occurred.

For established biofilms, 24 h old biofilms of *S. aureus* Xen29 were established as above. The washed biofilms were then exposed to 100 µL of NB1 containing 6 mg/mL of farnesol in a humidified container. After incubation at 37 °C for 0, 15, and 30 min, or 1, 2, 3, 4, and 24 h, media were removed and adherent biofilms were washed with 100 µL of PBS, then dislodged in 100 µL of PBS by vigorous (≥10 times) pipetting, and the CFU/mL of viable bacteria was determined by serial dilution and drop plating.

### 4.9. Propidium Iodide (PI) Influx Assay

Bacterial biofilms were established in plasma/collagen-pre-coated 96-well plates described as above. Washed biofilms were then exposed to 20 µM of PI (Invitrogen) at room temperature for 10 min, protected from light. Farnesol was then added into each well to reach various final concentrations in a total volume of 150 µL, and incubated at room temperature for 30 min, protected from light. The unbounded PI was then gently removed from each well and biofilms were washed with 150 µL of sterile water followed by the addition of 100 µL of sterile water into each well. PI fluorescence was then measured every 30 s for 5 min using a TECAN Infinite M200 microplate reader (Tecan, Männedorf, Switzerland). As a control, biofilms were exposed to the same amount of vehicle without farnesol.

### 4.10. Biofilm Detachment

To study potential biofilm detachment (besides cell killing) by farnesol, an intermediate (sub-lethal) concentration of farnesol was selected (0.1 mg/mL for biofilm formation; 1 mg/mL for established biofilm) for *S. aureus* Xen29. For biofilm formation, a fresh bacterial inoculum (1 × 10^6^ CFU/mL) was cultured in 100 µL of NB1/TSB containing farnesol (0.1 mg/mL for Xen29; 0.5 mg/mL for Xen5) in plasma-pre-coated 96-well plates in a humidified container. After 24 h incubation at 37 °C in a humidified container, planktonic cells were saved, biofilms were washed with 100 µL of PBS, then adherent biofilm cells were dislodged in 50 µL of PBS by vigorous (≥10 times) pipetting, and the CFU/mL of viable bacteria in both planktonic and biofilm populations were determined by serial dilution and drop plating. The total number of cells combined from both planktonic and biofilm sources, and the percentage of planktonic/biofilm cells compared to the total cells (for Xen29), or the ratios of planktonic vs. biofilm cells (for Xen5) were calculated. The lower limit of detection was 50 CFU/mL. To visualize the data on a logarithmic scale, a value of 50 CFU/mL was assigned when no growth occurred. Bacterial biofilms were established in plasma/collagen-pre-coated 96-well plates described as above. The washed biofilms were then exposed to 100 µL of NB1/TSB containing farnesol (1 mg/mL for Xen29; 0.2 mg/mL for Xen5). After an additional 24 h incubation in a humidified container, detached cells were saved, and adherent biofilm cells were washed with 100 µL of PBS, then dislodged with 100 µL of PBS using vigorous (≥10 times) pipetting, and the CFU/mL of viable bacteria was determined by serial dilution and drop plating. The total cells combined from both detached and biofilm cells, and the percentage of detached/biofilm cells compared to total cells (for Xen29), or the ratios of detached vs. biofilm cells (for Xen5), were calculated. As two negative controls, bacteria were cultured in media only or media containing the same amount of vehicle.

### 4.11. Infection and Treatment of Ex Vivo Human Skin

Human skin was obtained from healthy donors undergoing abdominoplasty in the Department of Plastic and Reconstructive Surgery at Wake Forest University School of Medicine, under an Institutional Review Board (IRB)-approved protocol. The human samples were de-identified, and classified as human waste, hence no informed consent was needed. None of the authors were involved in the tissue procurement. Skin samples were processed within an h after surgery, and excess subcutaneous fat was removed. Three-centimeter portions of skin were cut away and the epidermis was sprayed with 70% ethanol for 5 min, then soaked with sterile PBS (~50 mL/sample) for 10 min (total of four cycles of soaking to remove any potential blood, bacteria, or chemical residues). Burns were induced on the skin surface using a 3 cm diameter brass cylinder heated to 100 °C in 200 mM of polyethylene glycol solution for 10 s. The weight of the cylinder provided a consistent pressure (345 g) that was applied to the human skin during burn creation to induce a second-degree burn injury. The skin was then placed (epidermal side up) into one well of a 6-well plate containing 1 mL of Dulbecco’s modified eagle medium (DMEM, Gibco, Waltham, MA, USA) complemented with 2 mM of glutamine and 10% heat-inactivated fetal bovine serum (HI-FBS, Gibco). Only the lower dermis of the skin was immersed in the medium. Half of the soaked skin samples were retained as intact/unburned controls.

For skin infection experiments, 50 µL of a bacterial inoculum with various concentrations of farnesol [1 × 10^6^ CFU/mL (for Xen29) or 1 × 10^5^ CFU/mL (for Xen5) to evaluate the inhibition of biofilm formation, or 1 × 10^8^ CFU/mL (for Xen29) or 1 × 10^7^ CFU/mL (for Xen5) for established biofilms] was evenly distributed onto the intact or burned skin. The plates were incubated at 37 °C and 5% CO_2_ for 24 or 48 h in a humid chamber. Skin samples were then imaged by an in vivo imaging system (IVIS, PerkinElmer, Waltham, MA, USA). For treatment of 24 h old established biofilms, 100 µL of farnesol in medium was evenly distributed onto the established biofilms on skin surfaces and then incubated at 37 °C and 5% CO_2_ for an extra 24 h. The samples were then imaged as above. As a control, samples were exposed to the vehicle without farnesol.

To assess the viable bacterial count, three 5 mm punch biopsies were collected from the center of the skin. Bacteria obtained from the biopsy surface were collected by sterile swabbing (≥10 times) and mopped (≥20 times) thoroughly (with rotation of the swab) into a tube containing 1 mL of sterile PBS. Bacterial removal was confirmed by a luminometer showing of ≥ 95% reduction of luminescence intensity for the positive (vehicle) controls. The swab head with bacteria was cut off and dipped into the tube which was then vortexed thoroughly for 30 s. The number of viable bacteria (CFU/cm^2^ of skin surface) was determined by serial dilution and drop plating. The lower limit of detection was 255 CFU/cm^2^. To visualize the data on a logarithmic scale, a value of 255 CFU/cm^2^ was assigned when no growth occurred. An additional 5 mm punch biopsy was also collected and then fixed in 10% formalin, embedded in paraffin, 5-µm sectioned, and stained with hematoxylin and eosin (H&E stain) for histological examination. The stained sections were then imaged using a Zeiss Axioscope microscope (Zeiss, Oberkochen, Baden-Württemberg, Germany).

### 4.12. MTS Assay

CellTiter 96^®^ AQueous One Solution Cell Proliferation (MTS) assay (Promega, Madison, WI, USA) was used to determine the viability of HEKa (ATCC) when exposed to farnesol. Cells (5000/cm^2^) were cultured with 200 µL of keratinocyte-serum free medium (Gibco) in a collagen-coated 96-well plate to form a monolayer (≥70% of confluence) in a 37 °C, 5% CO_2_, humidified incubator. The medium was aspirated, cells were washed with 200 µL of PBS, and then incubated with 200 µL of medium containing 1, 6, or 15 mg/mL of farnesol. Cells were exposed to medium only, or medium containing the same amount of vehicle without farnesol as medium and vehicle controls. After 24 h incubation, the medium was aspirated, and the attached cells were washed with 200 µL of PBS, followed by the addition of 100 µL of MTS solution which had been diluted 1:4 with medium. The plate was incubated in the 37 °C, 5% CO_2_, humidified incubator for 2–4 h, while protected from light. The optical density at 490 nm was then measured using a TECAN Infinite M200 microplate reader with the MTS solution alone as a blank.

### 4.13. Statistical Analysis

Data are expressed as mean ± standard deviation of the mean unless stated otherwise. Statistical analysis of the data was performed using GraphPad Prism 9 (version 9.2.0). All statistical tests were two-sided. For analysis of means of three or more groups, analysis of variance (ANOVA) tests were performed. In the event that ANOVAs justified post hoc comparisons between group means, the comparisons were conducted using Tukey’s multiple comparisons test. Unpaired Student’s t-tests were used for comparisons between the means of two groups. The results were considered statistically significant at a value of *p* < 0.05.

## Data Availability

The datasets generated during and/or analyzed during the current study are available from the corresponding author on reasonable request.

## Supplementary Materials

The following supporting information can be downloaded at https://www.mdpi.com/article/10.3390/antibiotics13040350/s1, Figure S1. *S. aureus* Xen29 biofilm images shown in Figure 1C,D were quantitatively analyzed by Photoshop^®^ for fluorescence intensity and Comstat2 for biomass and average thickness of biofilms. Figure S2. Farnesol kills *S. aureus* Xen40 cells to inhibit its biofilm formation. Figure S3. Farnesol kills biofilm-encased cells of *S. aureus* Xen40, causing biofilm perforation and destruction. Figure S4. *P. aeruginosa* Xen5 biofilm images shown in Figure 2C,D were quantitatively analyzed by Photoshop^®^ for fluorescence intensity, and by Comstat2 for biomass and average thickness of biofilms. Figure S5. *P. aeruginosa* PAO1 biofilm images shown in Figure 2G,H were quantitatively analyzed by Photoshop^®^ for fluorescence intensity, and by Comstat2 for biomass and average thickness of biofilms. Figure S6. Farnesol is bactericidal against both *S. aureus* and *P. aeruginosa* as measured by propidium iodide influx. Figure S7. Farnesol protects from severe necrosis and easy peeling of human epidermis caused by the established biofilm infections of *P. aeruginosa* Xen5. Figure S8. Formula for conversion between OD600 and CFU/mL for the four *S. aureus* and *P. aeruginosa* strains used in the study. Table S1. Stocks of farnesol as an anti-bacterial agent in publications.
