# Supplementary material for: Repurposing Farnesol for Combating Drug-Resistant and Persistent Single and Polymicrobial Biofilms
Source: Antibiotics (Basel). 2024 Apr 11;13(4):350. doi: 10.3390/antibiotics13040350 (PMC11047559; doi:10.3390/antibiotics13040350)
Supplement: Supplementary file 1 [file antibiotics-13-00350-s001.zip › antibiotics-2950262-supplementary.pdf]

Table S1. Stocks of farnesol as an anti-bacterial agent in publications.

Source of farnesol	Isomers	Vehicle/solvent	Highest dose used	Reference
Cayman Chemical, USA	Mixture	Ethanol	15 mg/mL = 67.4 mM	This study
Adamas Reagent, China	Mixture	Unspecified	5 mM	[1]
Dargoco, Germany	Unspecified	Tween 80	2 mg/mL	[2,3]
Extrasynthese, France	Trans-trans	20% ethanol+0.75% dimethyl sulfoxide (DMSO)	1.33 mM	[4]
Extrasynthese, France	Trans-trans	25% ethanol+1.25% DMSO	5 mM	[5]
Sigma, Brazil	Trans-trans	Unspecified	0.3 mM	[6]
Sigma, USA	Trans-trans	15% ethanol+2.5% DMSO	1 mM	[7]
Sigma, USA	Trans-trans	20% ethanol+0.75% DMSO	1.4 mg/mL	[8]
Sigma, USA	Trans-trans	80% ethanol+20% DMSO	1.33 mM	[9]
Sigma, USA	Trans-trans	DMSO	0.2 mM	[10]
Sigma, USA	Trans-trans	DMSO	0.25 mM	[11]
Sigma, USA	Trans-trans	DMSO	0.5 mM	[12]
Sigma, USA	Trans-trans	15% ethanol	4 mM	[13]
Sigma, USA	Trans-trans	Ethanol	0.14 mM	[14]
Sigma, USA	Trans-trans	7.5% methanol	12.5 mM	[15]
Sigma, USA	Trans-trans	Methanol	0.2 mM	[16]
Sigma, USA	Trans-trans	Methanol	1.024 mM	[17]
Sigma, USA	Trans-trans	Unspecified	1 mg/ml	[18]
Sigma, USA	Trans-trans	Unspecified	0.3 mM	[19]
Sigma, USA	Trans-trans	Unspecified	30 mM	[20]
Sigma, USA	Unspecified	Methanol	0.05 mM	[21]
Sigma, USA	Unspecified	Methanol	0.3 mM	[22,23]
Sigma, USA	Unspecified	Unspecified	0.3 mM	[24,25]
Sigma, USA	Unspecified	Unspecified	0.08 mg/mL	[26]
Sigma, USA	Unspecified	Unspecified	2 mg/mL	[27]
Unspecified	Trans-trans	1% Tween 80	2.048 mg/mL	[28]
Wako, Japan	Unspecified	1% Tween 80	4 mg/mL	[29]

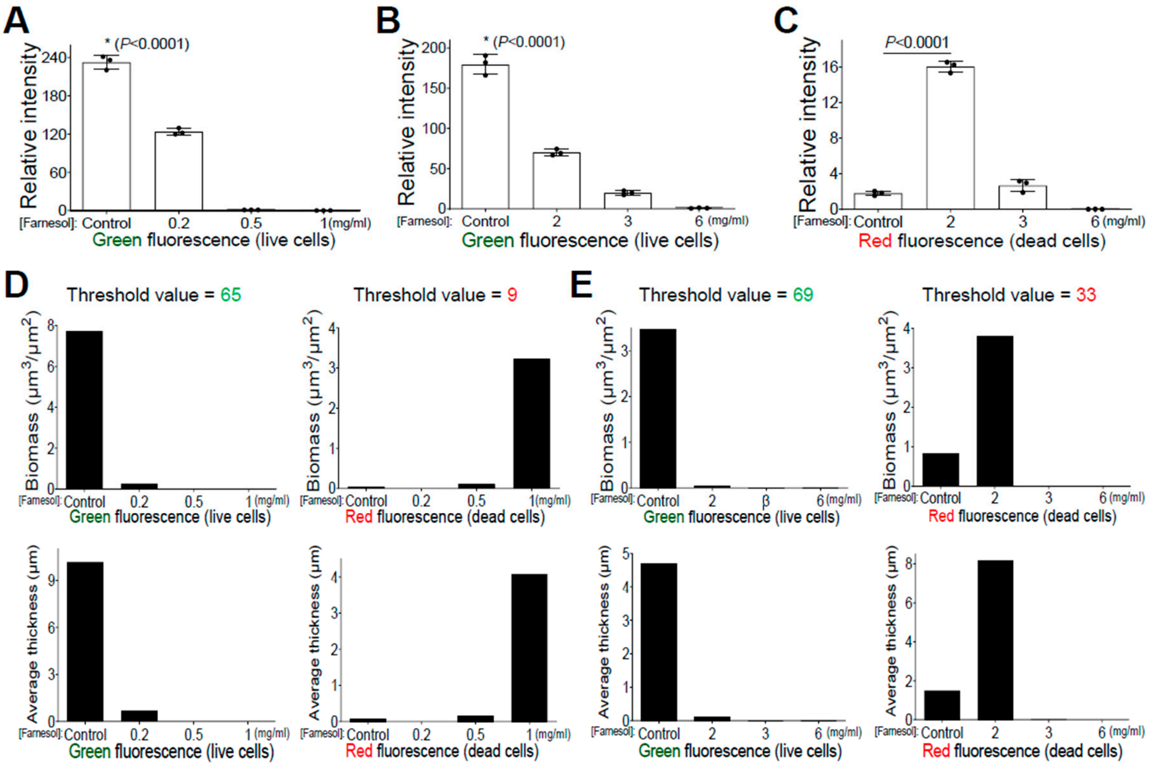

Figure S1. *S. aureus* Xen29 biofilm images shown in Fig. 1C and 1D were quantitatively analyzed by Photoshop® for fluorescence intensity and Comstat2 for biomass and average thickness of biofilms. **A, B, C,** Quantitative analysis of relative intensity of green fluorescence in the biofilm images shown in Fig. 1C (A), and green (B) or red (C) fluorescence in the biofilm images shown in Fig. 1D, by Photoshop® for fluorescence intensity. Data are shown as mean ± SD (n=3). The intensity of red fluorescence in the biofilm images shown in Fig. 1C was too weak to be detected by Photoshop®. **D, E,** Quantitative analysis of three-dimensional biofilm structure in the biofilm images shown in Fig. 1C (D) and Fig. 1D (E) by Comstat2, including biomass ($\mu m^3/\mu m^2$) and average thickness (μm). Threshold values for the analysis were set by Comstat2 using Otsu thresholding [30] in the images with the strongest green/red fluorescence and are shown at the top of the panels. The obtained threshold values were then applied to both control and treatment groups for fair comparison.

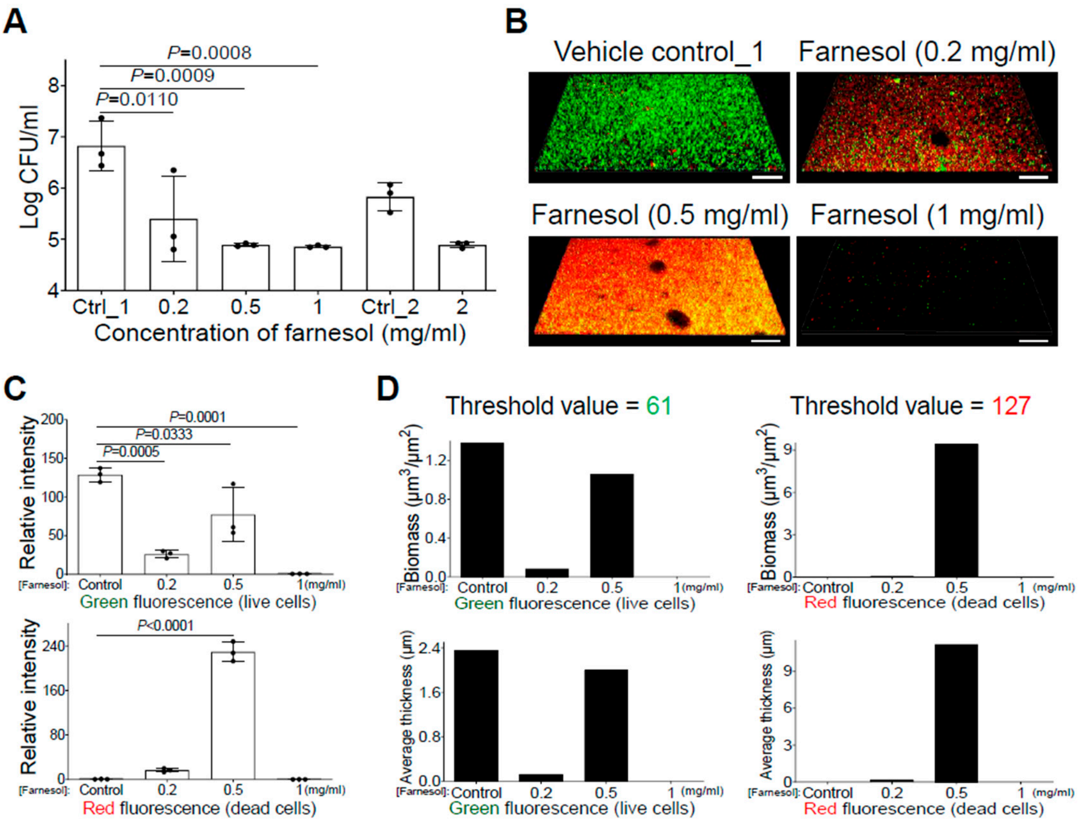

Figure S2. Farnesol kills *S. aureus* Xen40 cells to inhibit its biofilm formation. **A,** Inhibition of biofilm formation of *S. aureus* Xen40 by farnesol 24 h after incubation in NB1 in plasma-pre-coated wells. Results are expressed as the number of viable bacteria in log_{10} CFU per mL. Data are shown as mean ± SD (n=3). **B,** Three-dimensional merged images of Live/Dead viability of *S. aureus* Xen40 biofilms after 24-h incubation in NB1 containing farnesol. Biofilms were stained with both SYTO® 9 (green fluorescence for live cells) and propidium iodide (red fluorescence for dead cells). Scale bars, 20 μm. **C,** Quantitative analysis of relative fluorescence intensity in the biofilm images shown in (B) by Photoshop®. Data are shown as mean ± SD (n=3). **D,** Quantitative analysis of three-dimensional biofilm structure in the biofilm images shown in (B) by Comstat2, including biomass ($\mu m^3/\mu m^2$) and average thickness (μm). Threshold values of green and red fluorescence for the analysis are shown at the top of the panels.

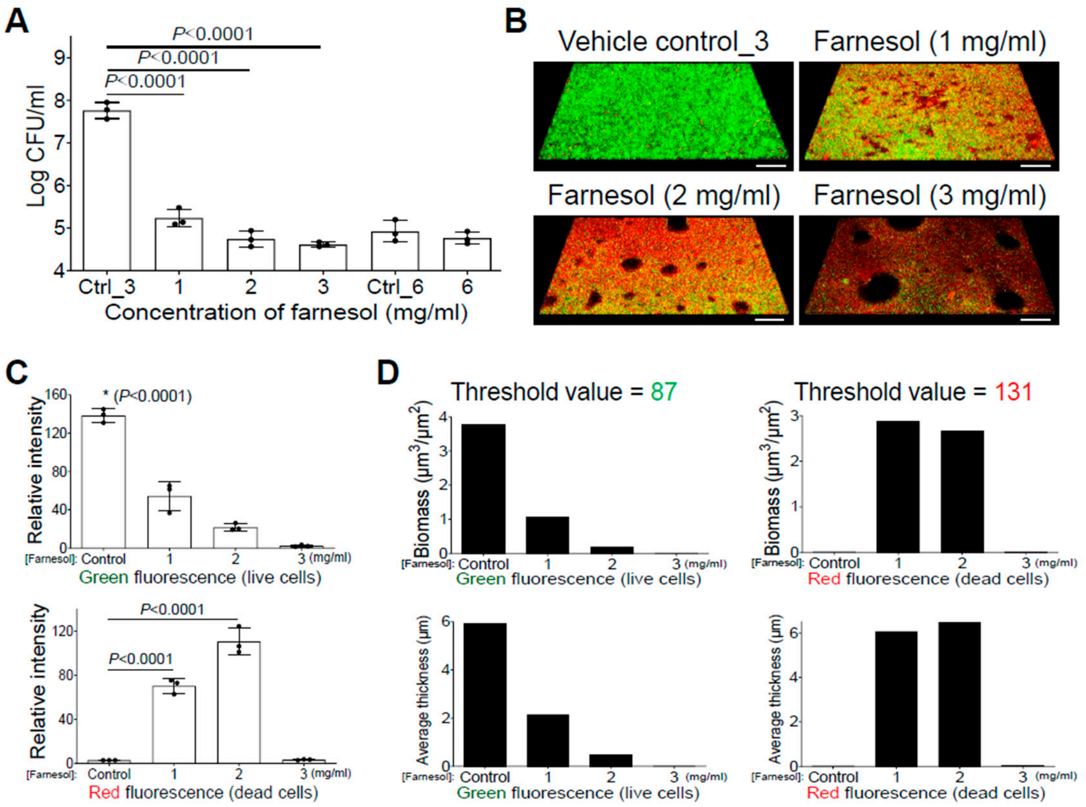

Figure S3. Farnesol kills biofilm-encased cells of *S. aureus* Xen40, causing biofilm perforation and destruction. A, Disruption of established biofilms of *S. aureus* Xen40 by farnesol. Results are expressed as the number of viable bacteria in $\log_{10}$ CFU per mL after 24-h exposure of 24-h-old established biofilms to farnesol. Data are shown as mean ± SD (n=3). **B,** Three-dimensional merged images of Live/Dead viability of *S. aureus* Xen40 biofilms after 24-h exposure of 24-h-old established biofilms in NB1 containing farnesol in plasma-precoated chambers. Biofilms were stained with both SYTO® 9 (green fluorescence for live cells) and propidium iodide (red fluorescence for dead cells). The presence of unstained hole-like areas in the biofilm matrix suggests biofilm perforation and destruction by farnesol. Scale bars, 20 μm. **C,** Quantitative analysis of relative fluorescence intensity in the biofilm images shown in (B) by Photoshop®. Data are shown as mean ± SD (n=3). **D,** Quantitative analysis of three-dimensional biofilm structure in the biofilm images shown in (B) by Comstat2, including biomass ($\mu m^3/\mu m^2$) and average thickness (μm). Threshold values of green and red fluorescence for the analysis are shown at the top of the panels.

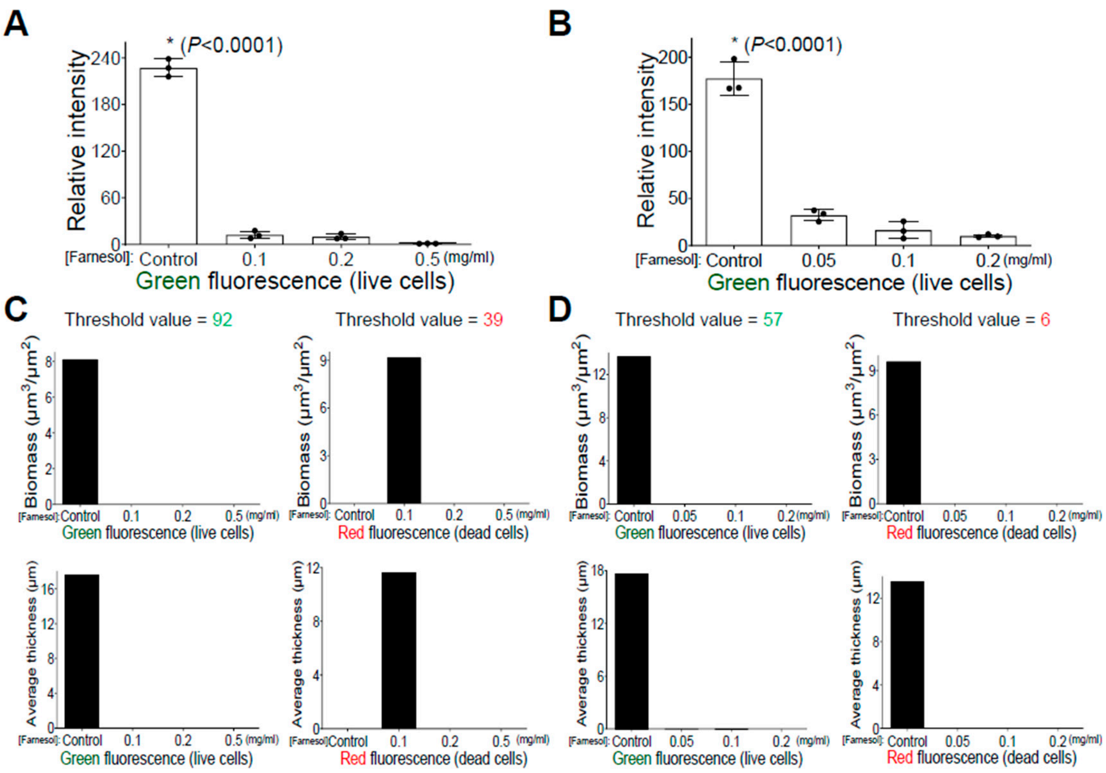

Figure S4. *P. aeruginosa* Xen5 biofilm images shown in Fig. 2C and 2D were quantitatively analyzed by Photoshop® for fluorescence intensity, and by Comstat2 for biomass and average thickness of biofilms. **A, B,** Quantitative analysis of relative green fluorescence intensity in the biofilm images shown in Fig. 2C (A) and Fig. 2D (B) by Photoshop®. Data were shown as mean ± SD (n=3). The intensity of red fluorescence in both biofilm images was too weak to be detected by Photoshop®. **C, D,** Quantitative analysis of three-dimensional biofilm structure in the biofilm images shown in Fig. 2C (C) and Fig. 2D (D) by Comstat2, including biomass ($\mu m^3/\mu m^2$) and average thickness (μm). Threshold values of green and red fluorescence for the analysis are shown at the top of the panels.

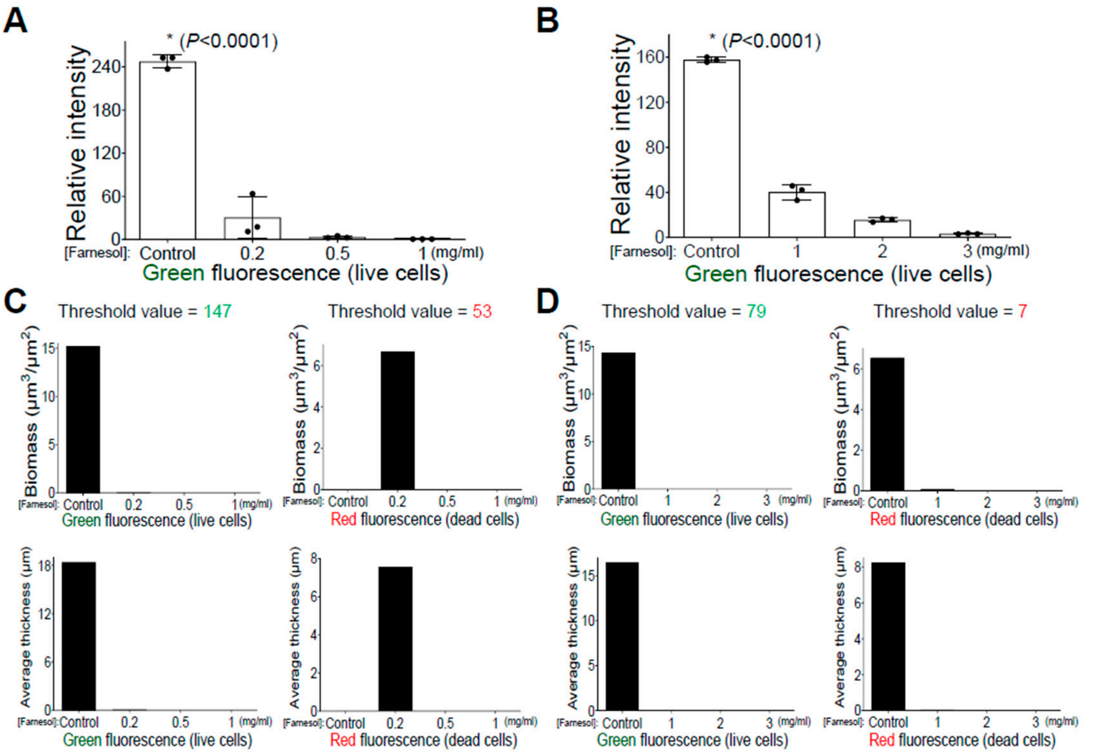

Figure S5. *P. aeruginosa* PAO1 biofilm images shown in Fig. 2G and 2H were quantitatively analyzed by Photoshop® for fluorescence intensity, and by Comstat2 for biomass and average thickness of biofilms. **A, B,** Quantitative analysis of relative green fluorescence intensity in the biofilm images shown in Fig. 2G (A) and Fig. 2H (B) by Photoshop®. Data are shown as mean ± SD (n=3). The intensity of red fluorescence in both biofilm images was too weak to be detected by Photoshop®. **C, D,** Quantitative analysis of three-dimensional biofilm structure in the biofilm images shown in Fig. 2G (C) and Fig. 2H (D) by Comstat2, including biomass ($\mu m^3/\mu m^2$) and average thickness (µm). Threshold values of green and red fluorescence for the analysis were shown at the top of the panels.

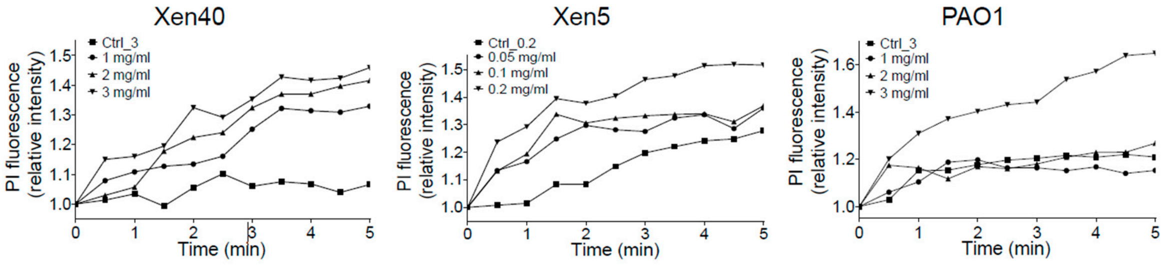

Figure S6. Farnesol is bactericidal against both *S. aureus* and *P. aeruginosa* as measured by propidium iodide influx. Data were normalized to the vehicle control (ethanol) at time zero and shown as the mean of three replicates.

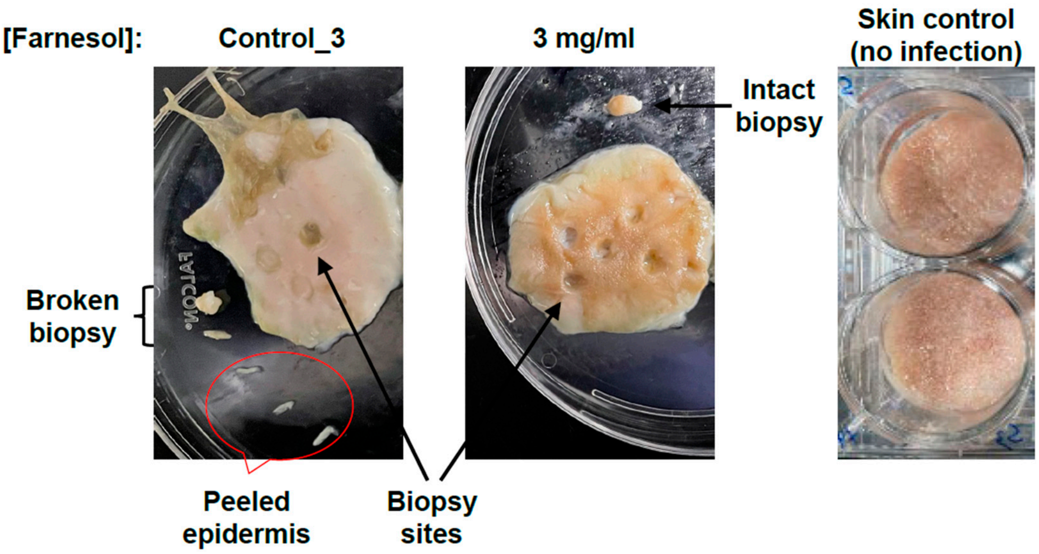

Figure S7. Farnesol protects from severe necrosis and easy peeling of human epidermis caused by the established biofilm infections of *P. aeruginosa* Xen5. The Xen5 infections caused severe damage on intact human skin so that the epidermis was discolored, and could be easily peeled away.

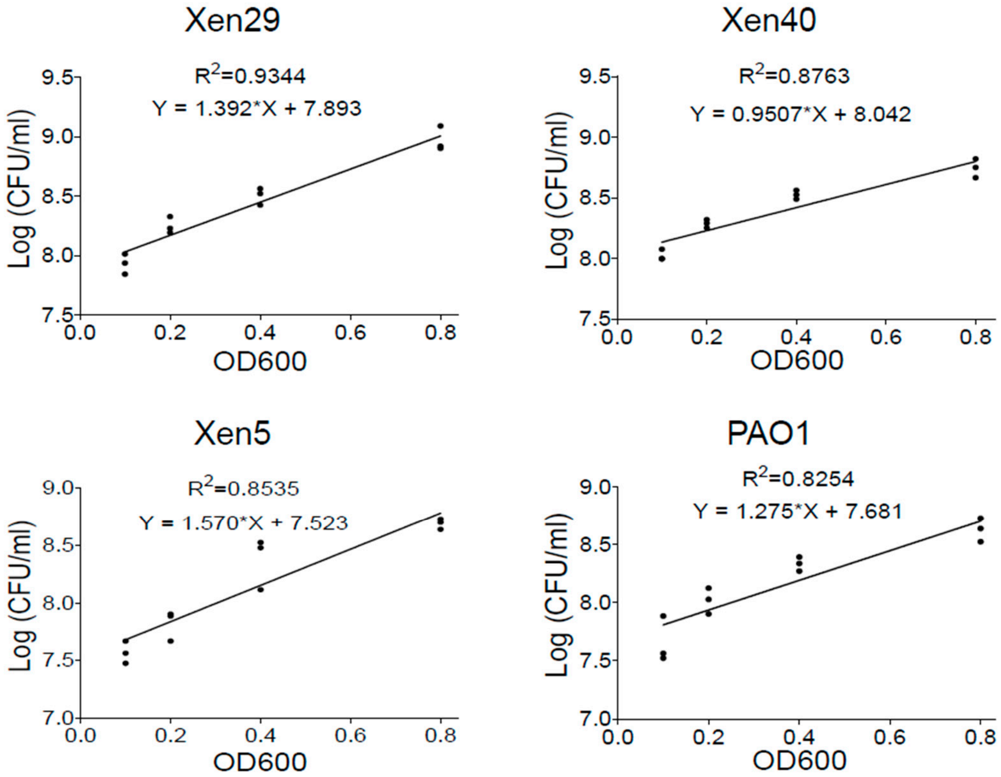

Figure S8. Formula for conversion between OD600 and CFU/ml for the four *S. aureus* and *P. aeruginosa* strains used in the study. *S. aureus* (Xen29 or Xen40) was cultured in the NB1 whereas *P. aeruginosa* (Xen5 and PAO1) was grown in the TSB. The effective linear range of OD600 is 0.1 to 0.8. The coefficient of determination (R^2) values for each formula are shown at the top of each panels.

References

1. Li, W.-R., Zeng, T.-H., Xie, X.-B., Shi, Q.-S. & Li, C.-L. Inhibition of the pqsABCDE and pqsH in the pqs quorum sensing system and related virulence factors of the Pseudomonas aeruginosa PAO1 strain by farnesol. *Int. Biodeterior. Biodegrad.* **151**, 104956 (2020).
2. Akiyama, H. *et al.* Actions of Farnesol and Xylitol against Staphylococcus aureus. *Chemotherapy* 7 **48**, 122–128 (2002).
3. Masako, K., Hideyuki, I., Shigeyuki, O. & Zenro, I. A novel method to control the balance of skin microflora: Part 1. Attack on biofilm of Staphylococcus aureus without antibiotics. *J. Dermatol. Sci.* **38**, 197–205 (2005).
4. Koo, H. *et al.* Inhibition of Streptococcus mutans biofilm accumulation and polysaccharide production by apigenin and tt-farnesol. *J. Antimicrob. Chemother.* **52**, 782–789 (2003).
5. Koo, H. *et al.* Apigenin and tt-Farnesol with Fluoride Effects on S. mutans Biofilms and Dental Caries. *J. Dent. Res.* **84**, 1016–1020 (2005).
6. Brilhante, R. S. N. *et al.* Sesquiterpene Farnesol Contributes to Increased Susceptibility to β-16 Lactams in Strains of Burkholderia pseudomallei. *Antimicrob. Agents Chemother.* **56**, 2198–2200 17 (2012).
7. Jeon, J.-G. *et al.* Influences of trans-trans farnesol, a membrane-targeting sesquiterpenoid, on Streptococcus mutans physiology and survival within mixed-species oral biofilms. *Int. J. Oral Sci.* **3**, 98–106 (2011).
8. De-Carli, A. D. *et al.* Antimicrobial activity of tt-farnesol associated with an endodontic sealer against 22 Enterococcus faecalis. *G. Ital. Endodonzia* **35**, (2021).
9. Koo, H. *et al.* Effects of apigenin and tt-farnesol on glucosyltransferase activity, biofilm viability and 24 caries development in rats. *Oral Microbiol. Immunol.* **17**, 337–343 (2002).
10. Hassan Abdel-Rhman, S., Mostafa El-Mahdy, A. & El-Mowafy, M. Effect of Tyrosol and Farnesol on Virulence and Antibiotic Resistance of Clinical Isolates of Pseudomonas aeruginosa. *BioMed Res. Int.* **2015**, e456463 (2015).
11. Cugini, C. *et al.* Farnesol, a common sesquiterpene, inhibits PQS production in Pseudomonas aeruginosa. *Mol. Microbiol.* **65**, 896–906 (2007).
12. Pammi, M., Liang, R., Hicks, J. M., Barrish, J. & Versalovic, J. Farnesol Decreases Biofilms of Staphylococcus epidermidis and Exhibits Synergy With Nafcillin and Vancomycin. *Pediatr. Res.* **70**, 578–583 (2011).
13. Rocha, G. R., Florez Salamanca, E. J., de Barros, A. L., Lobo, C. I. V. & Klein, M. I. Effect of tt-farnesol and myricetin on in vitro biofilm formed by Streptococcus mutans and Candida albicans. *BMC Complement. Altern. Med.* **18**, 61 (2018).
14. Kossakowska-Zwierucho, M., Szewczyk, G., Sarna, T. & Nakonieczna, J. Farnesol potentiates photodynamic inactivation of Staphylococcus aureus with the use of red light-activated porphyrin TMPyP. *J. Photochem. Photobiol. B* **206**, 111863 (2020).
15. Fernandes, R. A. *et al.* Virulence Factors in Candida albicans and Streptococcus mutans Biofilms Mediated by Farnesol. *Indian J. Microbiol.* **58**, 138–145 (2018).
16. Vila, T. *et al.* Candida albicans quorum-sensing molecule farnesol modulates staphyloxanthin production and activates the thiol-based oxidative-stress response in Staphylococcus aureus. *Virulence* **10**, 625–642 (2019).
17. Cao, L., Zhang, Z., Xu, S., Ma, M. & Wei, X. Farnesol inhibits development of caries by augmenting oxygen sensitivity and suppressing virulence-associated gene expression inStreptococcus mutans. *J. Biomed. Res.* **31**, 333–343 (2017).
18. Lopes, A. P. *et al.* Antimicrobial, modulatory, and antibiofilm activity of tt-farnesol on bacterial and fungal strains of importance to human health. *Bioorg. Med. Chem. Lett.* **47**, 128192 (2021).
19. Cerca, N. *et al.* Farnesol induces cell detachment from established S. epidermidis biofilms. *J. Antibiot. (Tokyo)* **66**, 255–258 (2013).
20. Unnanuntana, A., Bonsignore, L., Shirtliff, M. E. & Greenfield, E. M. The Effects of Farnesol on Staphylococcus aureus Biofilms and Osteoblasts. *J. Bone Joint Surg. Am.* **91**, 2683–2692 (2009).
21. Kong, E. F., Tsui, C., Kucharíková, S., Van Dijck, P. & Jabra-Rizk, M. A. Modulation of Staphylococcus aureus Response to Antimicrobials by the Candida albicans Quorum Sensing Molecule Farnesol. *Antimicrob. Agents Chemother.* **61**, e01573-17 (2017).
22. Gomes, F. I. A., Teixeira, P., Azeredo, J. & Oliveira, R. Effect of Farnesol on Planktonic and Biofilm Cells of Staphylococcus epidermidis. *Curr. Microbiol.* **59**, 118–122 (2009).
23. Gomes, F., Teixeira, P., Cerca, N., Azeredo, J. & Oliveira, R. Effect of Farnesol on Structure and Composition of Staphylococcus epidermidis Biofilm Matrix. *Curr. Microbiol.* **63**, 354 (2011).
24. Cerca, N., Gomes, F., Pereira, S., Teixeira, P. & Oliveira, R. Confocal laser scanning microscopy analysis of S. epidermidis biofilms exposed to farnesol, vancomycin and rifampicin. *BMC Res. Notes* **5**, 244 (2012).
25. Jabra-Rizk, M. A., Meiller, T. F., James, C. E. & Shirtliff, M. E. Effect of Farnesol on Staphylococcus 64 aureus Biofilm Formation and Antimicrobial Susceptibility. *Antimicrob. Agents Chemother.* **50**, 1463–1469 (2006).
26. Kaneko, M., Togashi, N., Hamashima, H., Hirohara, M. & Inoue, Y. Effect of farnesol on mevalonate 67 pathway of Staphylococcus aureus. *J. Antibiot. (Tokyo)* **64**, 547–549 (2011).

27. Alves, F. R. F., Neves, M. A. S., Silva, M. G., Rôças, I. N. & Siqueira Jr., J. F. Antibiofilm and Antibacterial Activities of Farnesol and Xylitol as Potential Endodontic Irrigants. *Braz. Dent. J.* **24**, 224–229 (2013).
28. Kim, C., Hesek, D., Lee, M. & Mobashery, S. Potentiation of the activity of β-lactam antibiotics by farnesol and its derivatives. *Bioorg. Med. Chem. Lett.* **28**, 642–645 (2018).
29. Kuroda, M., Nagasaki, S. & Ohta, T. Sesquiterpene farnesol inhibits recycling of the C55 lipid carrier of the murein monomer precursor contributing to increased susceptibility to β-lactams in methicillin-resistant Staphylococcus aureus. *J. Antimicrob. Chemother.* **59**, 425–432 (2007).
30. Otsu, N. A Threshold Selection Method from Gray-Level Histograms. *IEEE Trans. Syst. Man Cybern.* **1979**, *9*, 62–66. https://doi.org/10.1109/TSMC.1979.4310076.
